# Time-resolved structural evolution during the collapse of responsive hydrogels: The microgel-to-particle transition

**DOI:** 10.1126/sciadv.aao7086

**Published:** 2018-04-06

**Authors:** Rico Keidel, Ali Ghavami, Dersy M. Lugo, Gudrun Lotze, Otto Virtanen, Peter Beumers, Jan Skov Pedersen, Andre Bardow, Roland G. Winkler, Walter Richtering

**Affiliations:** 1 Chair of Technical Thermodynamics, Rheinisch-Westfälische Technische Hochschule (RWTH) Aachen University, Schinkelstrasse 8, 52062 Aachen, Germany.; 2 Institute for Advanced Simulation, Forschungszentrum Jülich, 52425 Jülich, Germany.; 3 Institute of Physical Chemistry, RWTH Aachen University, Landoltweg 2, 52056 Aachen, Germany.; 4 European Synchrotron Radiation Facility (ESRF), ID02–Time-Resolved Ultra Small-Angle X-Ray Scattering, 71 Avenue des Martyrs, CS 40220, 38043 Grenoble Cedex 9, France.; 5 Interdisciplinary Nanoscience Center (iNANO) and Department of Chemistry, Aarhus University, 8000 Aarhus, Denmark.; 6 Institute of Energy and Climate Research: Energy Systems Engineering (IEK-10), Forschungszentrum Jülich, 52425 Jülich, Germany.; 7 DWI–Leibniz Institute for Interactive Materials, Forckenbeckstrasse 50, D-52056 Aachen, Germany.

## Abstract

Adaptive hydrogels, often termed smart materials, are macromolecules whose structure adjusts to external stimuli. Responsive micro- and nanogels are particularly interesting because the small length scale enables very fast response times. Chemical cross-links provide topological constraints and define the three-dimensional structure of the microgels, whereas their porous structure permits fast mass transfer, enabling very rapid structural adaption of the microgel to the environment. The change of microgel structure involves a unique transition from a flexible, swollen finite-size macromolecular network, characterized by a fuzzy surface, to a colloidal particle with homogeneous density and a sharp surface. In this contribution, we determine, for the first time, the structural evolution during the microgel-to-particle transition. Time-resolved small-angle x-ray scattering experiments and computer simulations unambiguously reveal a two-stage process: In a first, very fast process, collapsed clusters form at the periphery, leading to an intermediate, hollowish core-shell structure that slowly transforms to a globule. This structural evolution is independent of the type of stimulus and thus applies to instantaneous transitions as in a temperature jump or to slower stimuli that rely on the uptake of active molecules from and/or exchange with the environment. The fast transitions of size and shape provide unique opportunities for various applications as, for example, in uptake and release, catalysis, or sensing.

## Teaser

The collapse kinetics of microgels is determined experimentally and by mesoscale computer simulations.

## INTRODUCTION

Adaptive polymers, often termed smart materials, are macromolecules whose structure adjusts to external stimuli such as temperature, pH, solvent composition, or the uptake or release of molecules (*1*). Hydrogels are a distinct type of adaptive polymers because they allow for the preparation of responsive materials within natural biological environments. Typically, a stimulus induces a volume change of the hydrogel, which is connected to changes of other physical properties, for example, optical and mechanical. Thus, hydrogels are used, for example, as soft actuators, such as for small-scale robotics (*2*, *3*). Proper functionalization renders hydrogels sensitive to chemical signals, which can be exploited for drug release and sensing (*4*–*6*). A particular class of gels are so-called micro- or nanogels, with dimensions in the colloidal-size range. They are of special interest because they provide volume changes at small dimensions or even at local compartments (*7*) and are used in a variety of applications as, for example, in catalysis, separation technology, sensors, and medicine (*8*–*15*). Microgels are able to, for example, spread at interfaces or translocate through small pores and even cross the blood-brain barrier to release medication exactly where needed (*16*–*18*).

All these applications typically rely on the fast adaptation of the hydro-microgels. The response of the (hydro)gel to an external (chemical) trigger depends on the mass transport of the trigger into the gel and (part) of solvent out of the gel. Obviously, this mass transport is especially relevant for the response kinetics, and gel porosity and size play an important role (*19*–*21*). Thus, for the rational design of functional microgels, it is important to unravel their structural evolution upon a stimulus and, specifically, to identify the rate-determining processes. This is particularly interesting for the stability of absorbed substances and their release, for example, in drug delivery.

From a fundamental point of view, the process of volume change of microgels is related to two distinct colloidal stable states: (i) a collapsed conformation, with a homogeneous density and a sharp surface; and (ii) a swollen state, where microgels are soft, deformable networks with a fuzzy surface; here, the cross-links provide topological constraints for the network chains and enable compartmentalization. Currently, it is not known what the time scales for the volume change of microgels are and what structure the microgels assume during a collapse transition.

Here, we present experimental and simulation results for the evolution of the microgel structure upon solvent exchange. In experiments, we adopt the well characterized poly(*N*-isopropylacrylamide) (PNIPAM) microgels. On the one hand, this is a well established model system (*22*), and on the other hand, it resembles the behavior of proteins (*23*, *24*) and is used in many fabrications of biohybrid materials (*25*). In particular, we exploit the sensitivity of PNIPAM to the composition of water-methanol mixtures. Both solvents act as so-called good solvents and leave the microgels in a swollen state (for temperatures lower than the volume phase transition temperature). If the second solvent, referred to as cononsolvent, is added, then microgels collapse in a mixture of H_2_O/MeOH [with the most pronounced collapse at approximately 20 mole percent (mol %) of MeOH]. This effect is called cononsolvency (*26*, *27*). Precise mechanisms leading to a collapse and a slight preferential partitioning of one solvent species inside the gel are still under controversial debate (*28*–*33*). These details, however, will be mainly relevant on a local length scale and thus on a very short time scale, whereas the process of gel collapse concerns much larger length and longer time scales and hence can be considered as generic, which is also supported by our results discussed below.

To study the structural evolution, we dissolved microgels in pure H_2_O or methanol (MeOH), respectively, and rapidly changed the solvent composition in the vicinity of microgels. The associated mass transport into and out of the microgel leads to a modification of the internal solvent composition and induces a volume change of the microgel.

Although the equilibrium structure of microgels in the swollen and collapsed state has been investigated for a variety of systems [for example, see the studies of Stuart *et al*. (*1*), Brown *et al*. (*12*), Lu and Ballauf (*15*), Bischofberger *et al*. (*32*), Yang and Zhao (*33*), Maccarrone *et al*. (*34*), Stieger *et al*. (*35*), Berndt *et al*. (*36*), Stieger *et al*. (*37*), and Maccarrone *et al*. (*38*)], there are only a few studies investigating the volume change kinetics during transitions between these states (*39*–*47*). The experimental investigation of the collapse kinetics is challenging because of the small microgel size and the very short time scales involved. The latter requires a measurement technique capable of resolving these short time scales. The few experiments available in the literature determine a volume change (triggered by temperature, pH, and glucose) by means of turbidity and/or scattered light intensity (at a single scattering angle) (*39*–*47*). These data are correlated with the microgel size and thus reflect its volume-change kinetics. However, quantitative information on the size and the internal structure during the collapse transition cannot be inferred from these measurements.

We determine the evolution of the microgel structure during the transition from a swollen to a collapsed state by two approaches. First, experimentally, by means of time-resolved small-angle x-ray scattering (TR-SAXS), covering a broad range of scattering angles, which enables a quantitative analysis of the time-dependent microgel structure (*48*). The solvent exchange is achieved with a stopped-flow device. The microgel, dissolved in one solvent, is rapidly mixed with the cononsolvent such that the final solvent mixture contains 20 mol % of methanol. In particular, we study the two pathways, from both pure solvents (H_2_O and MeOH) to a solvent mixture of 20 mol % of MeOH. Second, theoretically, by mesoscale hydrodynamic computer simulations allowing for a systematic investigation of the key parameters governing the collapse while providing structural insight during the collapse process. Specifically, we exploit the multiparticle collision dynamics (MPC) approach for the fluid combined with molecular dynamics simulations for the polymers (*49*, *50*). The MPC method captures thermal fluctuations and has been shown to correctly account for hydrodynamic interactions (HIs) (*51*), that is, it captures fluid-mediated interactions and reproduces the hydrodynamic properties of polymers in solution (*51*), the dynamics of which is typically accelerated by the emerging flow fields of fluctuating or dragged monomers.

Computer simulations with and without HIs have previously been performed to study the collapse kinetics of linear polymers. Without HIs, simulations reveal a coil-to-globule transition proceeding through an initial formation of localized blobs along the chain followed by a crumpling stage, where the blobs grow and merge together, forming a collapsed globule (*52*, *53*). HIs not only change the coil-to-globule transition by the emergence of different intermediate states but also accelerate the collapse process substantially (*54*–*56*).

To the best of our knowledge, no time-resolved experimental study of the collapse kinetics of microgels has been presented so far nor have computer simulations been performed accounting for HIs. As we will outline, HIs are essential to explain the experimentally measured collapse kinetics.

By a combination of data from experiments and simulations, we address the following points: (i) We resolve the structural changes of a microgel from an open, porous network to a dense colloid—a piece of information essential for many applications. (ii) From time-resolved SAXS, we explicitly obtain precise data on the size and structure of a microgel during the collapse induced by cononsolvency. (iii) By computer simulations, we identify the rate-determining steps during the collapse transition. (iv) The computer simulations reveal the generic features of a collapse transition independent of the type of trigger.

## RESULTS

We observed the volume change process by combining the stopped-flow technique with TR-SAXS measurements. The structures of the microgel in the equilibrium initial state (pure methanol) and in the final state (water-methanol mixture containing 20 mol % of methanol) were investigated independently. In the initial state, the microgel reveals a fuzzy structure with a smoothly decaying segment density at the periphery (*37*) and a hydrodynamic radius of 954 nm. In the final state, the microgel forms a homogeneously collapsed sphere with a radius of 330 nm.

### Scattering curves

**Fig. 1:**
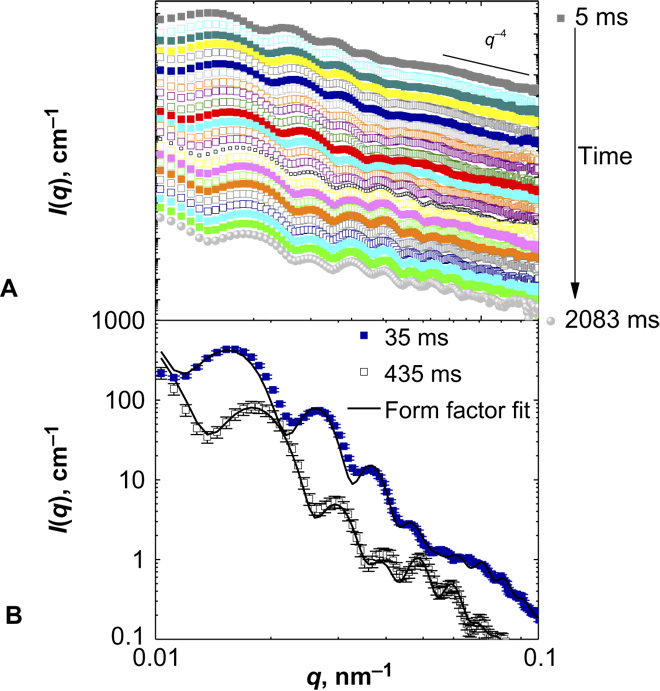
SAXS curves. (**A**) SAXS patterns of PNIPAM microgels for the solvent composition change from pure MeOH to *x*_MeOH_ = 0.20 at different times. (**B**) Examples of form factor fits for two SAXS patterns obtained at 35 and 435 ms after mixing (for better visibility, the intensity of the SAXS pattern at 435 ms is shifted vertically by a factor of 0.3).

Figure 1A shows the temporal evolution of the SAXS data after the solvent exchange from pure MeOH to the methanol-water mixture. The scattering intensity curves are characterized by various minima reflecting the high monodispersity of the microgels. The minima shift to larger *q* values with time, directly revealing the collapse of the microgels. In the high *q* region, the intensity decreases proportional to *q*^−4^ and Porod behavior manifests the sharp particle surface (*37*).

The *q* dependence of the scattering profiles, that is, the form factor, provides insight into the structure (size and shape) of the particles and can be quantitatively analyzed (*57*–*62*). The positions of the form factor minima and maxima in the scattering pattern allow us to determine the microgel size. The scattering patterns recorded a long time after adding the cononsolvent reveal a pronounced first minimum and static light scattering (SLS; providing access to lower *q*) at the equilibrium state, proving that this is indeed the first minimum of the form factor (for details, see section S1.6). At shorter times, this first minimum is observed at lower *q* and is hardly visible at the shortest times. However, for the SAXS measurements shortly after the mixing, the first minimum can be consistently described with a form factor model that fits the entire scattering profiles as displayed in Fig. 1B.

The scattering curves differ with respect to the position and height of the minima and maxima, indicating internal structural changes of the microgels. Furthermore, the large number of minima and maxima allows for discrimination between different form factor models. Simple models, for example, of homogeneous spheres or spheres with core-shell structures, were not able to describe the experimental scattering curves over the entire *q* range (for details, see sections S1.9 and S1.10, where additional fits for various instances in time and for the solvent change starting from pure H_2_O are shown). Instead, the fitting of the scattering curve over the entire *q* range required a model of spheres with core-shell morphology and that further includes the presence of distributed small collapsed regions in a size range of 20 to 30 nm (*63*). We also tested a form factor model, with regions of higher density located at the periphery of the microgel; however, it did not lead to a better fit (see the Supplementary Materials), and because we have no other evidence of this layer, this model was abandoned. The model used thus assumes that local collapsed regions occur in the entire microgel, and because the collapse transition also gives the shell in the core-shell structure, we can conclude that the SAXS data agree with a collapse transition, which involves structural changes on two different length scales.

### Evolution of microgel structure

**Fig. 2:**
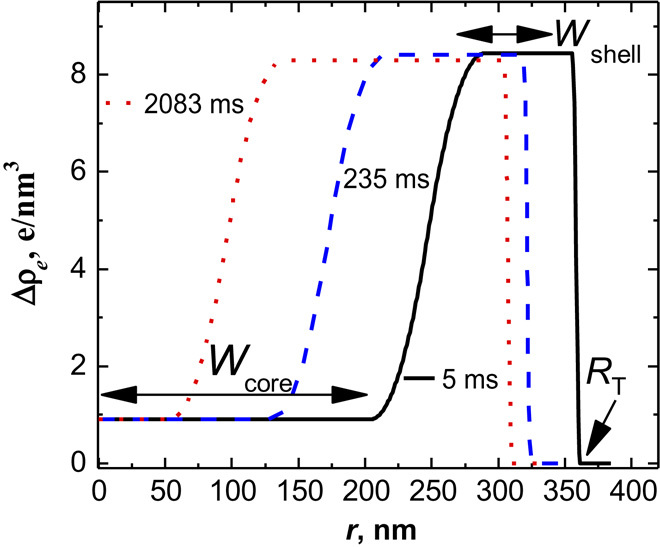
Radial excess electron density profiles calculated from the form factor model. The structural change of PNIPAM microgels for a solvent composition change starting from pure MeOH to *x*_MeOH_ = 0.2 in the MeOH/H_2_O mixture is presented for different times (5, 235, and 2083 ms).

**Fig. 3:**
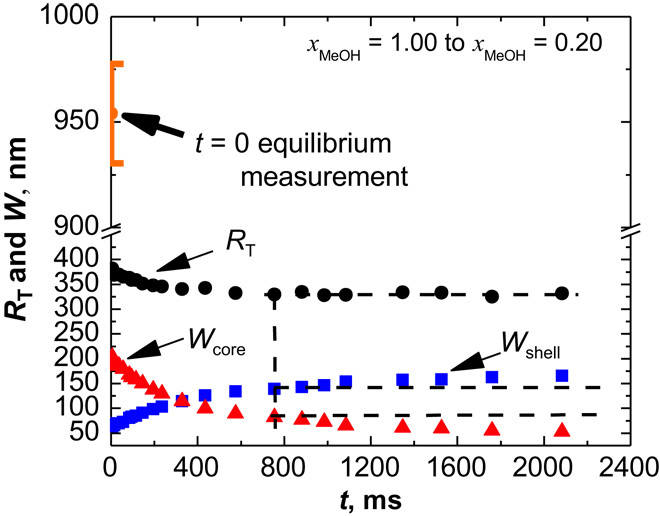
Microgel size and internal structure change from the form factor fit. The collapse transition of the PNIPAM microgel induced by the solvent composition change from pure MeOH to *x*_MeOH_ = 0.20 is shown. The evolution of the microgel size *R*_T_, of the core region *W*_core_, and of the shell *W*_shell_ is represented in black, red, and blue, respectively.

The form factor analysis allows us to determine radial excess electron density profiles. To model the form factor, a density profile is considered with a core (width *W*_core_), a shell (width *W*_shell_), and a transient region in between (cf. Fig. 2). The shell of collapsed polymers exhibits a sharp outer surface (Porod behavior) and the shell width increases as the total radius (*R*_T_) decreases slightly with time. Figure 3 depicts the time dependence of the total size of the microgel (*R*_T_), the width of the core region (*W*_core_), which decreases during the collapse, and the width of the high-density shell (*W*_shell_), which increases with time. Figure 3 suggests that the microgels collapse immediately upon the addition of the cononsolvent, as indicated by the jump from the initial size of 954 nm (*t* = 0) of the microgels to the slowly varying value of 381 nm at 5 ms, a rapid change that cannot be captured by the TR-SAXS experiments. Over the next 760 ms, the microgel slowly approaches the equilibrium radius of 331 nm. Hence, the density profiles (Fig. 2) correspond to a second, slower process, where the core-shell structure relaxes into a homogeneous sphere. As will be shown below, a similar behavior is found in our computer simulations, with a clear demonstration of the two processes. We would like like to emphasize here that the slow relaxation is not governed by cosolvent transport, as that penetrated the microgels during the fast, initial decay. Our experiments reveal a two-state process.

The literature indicates the presence of multiple processes (*43*, *44*). The fitting of our data to the time-dependent microgel size requires at least two exponential functions, corresponding to the two involved processes, and yields the time constants of τ_1_ = 1.3 ms and τ_2_ = 227 ms. An explanation for these two processes is provided by the computer simulations discussed below.

### Computer simulations

**Fig. 4:**
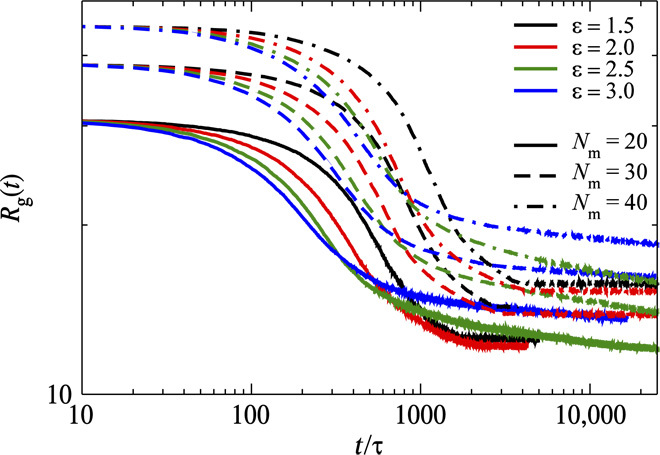
Simulation results of the time evolution of the microgel radius of gyration for systems with different quenching depths ε and different polymer lengths *N*_m_.

The microgel particle is embedded in an explicit solvent, which we model by the MPC approach (*49*). The solvent exchange is described implicitly by a change of the Lennard-Jones (LJ) attraction strength ε (quenching depth) between the monomers (for details, see section S2.2), that is, a single fluid is considered, which captures HIs. Results for the time evolution of the radius of gyration of microgels for different quenching depths ε and lengths *N*_m_ of the polymer network chains are presented in Fig. 4. Here, every monomer instantaneously experiences the change in solvent quality from good to bad solvent conditions. The results suggest that, for low ε values (that is, ε ≤ 2.0), microgels collapse until they reach a globular state. In contrast, for larger quenching depths, the microgels exhibit a two-step collapse: a fast collapse at short times followed by a slower decay for longer times. We would like to emphasize that this behavior is observed for all network chain lengths, despite the increasing relaxation time with increasing length. Qualitatively, the collapse speed in the first regime is directly proportional to the quenching depth, whereas in the slow regime, it is inversely proportional to ε. The time dependence of the radius of gyration of polymers during their collapse is usually described by the power law $\langle R_{g}^{2}(t)\rangle =\langle R_{g}^{2}(0)\rangle -At^{\alpha }$, with α ranging from 0.92 to 1.05 in the presence of HIs (*54*). The *R*_g_ values of Fig. 4 exhibit a similar power-law decay with the exponent α = 1.08 ± 0.05 (for details, see fig. S18). Hence, on the basis of the studies of linear polymers (*54*), where an exponent of approximately unity is found for collapsing linear polymers in the presence of HIs and a significantly smaller exponent without HIs, we conclude that the fast collapse is driven by HIs.

Figure 5 (a video is provided as supporting information) depicts the evolution of the radial monomer density for a microgel with *N*_m_ = 20 and the high quenching depth ε = 2.5. Immediately after the quench, that is, at *t* = 25τ, polymers start to form small clusters at cross-links, while the overall size of the microgel is essentially unaffected. Next, these clusters start to merge from the periphery of the microgel, and its size decreases strongly (*t* = 200τ). Eventually, the polymers in the core are also attracted and absorbed to the high-density periphery, forming a core-shell structure with a hollow core. For longer times, a slower process sets in, with a rearrangement and relaxation of the network chains due to strong attraction between monomers (*t* = 2000τ), and the microgel finally assumes a compact globular structure. The analysis of the thickness of the core, *W*_core_, and the shell, *W*_shell_, during the slow process reveals a faster dynamics of the inner shell surface, whereas the total size of the microgel, *R*_T_, decreases very slowly (for the definition of *W*_core_, *W*_shell_, and *R*_T_, see the Supplementary Materials). The comparably small variation of the overall radius of gyration compared to the core radius is consistent with the weak dependence of the inertia tensor of a core-shell sphere on the inner radius.

**Fig. 5:**
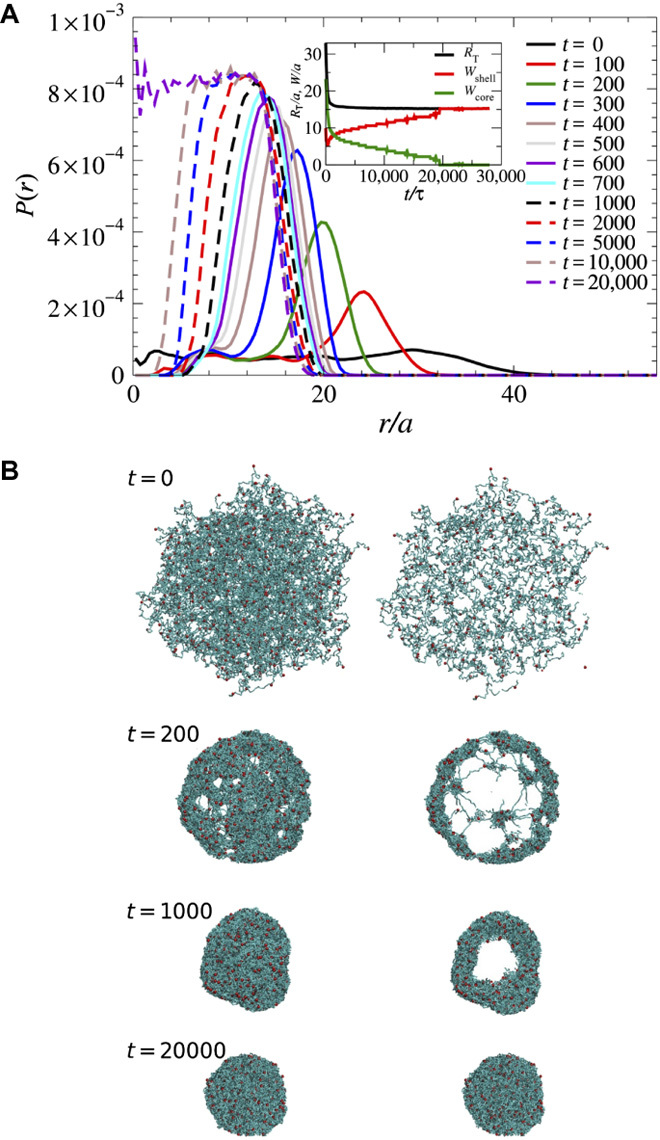
Simulation results of the time-dependent structural evolution of a microgel. (**A**) Radial monomer density distribution *P*(*r*) for a microgel with *N*_*m*_ = 20 and ε = 2.5. The various curves correspond to different times. The inset shows the time evolution of the outer radius *R*_T_, the shell thickness *W*_shell_, and the core thickness *W*_core_, respectively. (**B**) The snapshots illustrate the full microgel structure. (Left) Two-dimensional (2D) projection of the 3D monomer density and (right) 2D projection of monomers within a thin slice containing the center of mass of the microgel at indicated times during the simulation.

So far, we have considered an instantaneous change of the environment of a microgel, which leads to a specific collapse speed *v*_0_ depending on the quenching depth and microgel size. This transition corresponds, for example, to a change in temperature. However, in our experiments, the collapse is driven by a diffusive process (that is, cononsolvency), which requires the transport of solvents into and out of microgels, respectively. Such a transport can be modeled by a time- and position-dependent attraction strength ε. Here, we find that the initial fast collapse regime is diffusion-limited, when the transport velocity of the cononsolvent is slower than *v*_0_. However, the collapse becomes independent of solute diffusion for transport velocities larger than *v*_0_. The slow collapsing regime is preserved even for very slow cononsolvent transport if the quenching depth is sufficiently high. This suggests that the cononsolvent transport only affects the fast collapse, whereas the slow collapsing regime remains unaffected.

## DISCUSSION

We have quantitatively studied the structural evolution of microgel systems during the collapse transition through experiments and computer simulations. TR-SAXS provides structural insight into the microgel collapse transition from a swollen state, with polymer network characteristics, to a collapsed state, with colloidal properties. Two characteristic length scales are observed: small collapsed regions and a hollow core-shell structure; both also appear in the computer simulations. The analysis of TR-SAXS measurements reveals further structural changes after the microgel size reaches its equilibrium value (Fig. 3 at about 760 ms). The form factor is still different from that of a microgel at equilibrium (for details, see fig. S11). This is explained by our computer simulations, which show that the thickness of the shell increases faster than the overall microgel size decreases because of the rearrangement of polymer chains at the inner surface of the hollow microgel.

In the literature, so far, the microgel collapse has been studied experimentally through transmittance or scattering intensity measurements at a single scattering angle (*39*–*47*). These studies did not provide quantitative data on microgel size and structure; hence, a direct comparison of our experimental results with literature data is not possible. However, on the basis of our experimental and simulation results, we expect that details of a particular microgel system, for example, size, chemical structure of the polymer and cross-linker, and the type of external stimulus (temperature and pH jump), will not lead to fundamentally different behaviors. Thus, some interesting comparisons and interpretations of current results are possible. Temperature-jump experiments with PNIPAM microgels with a diameter of 100 to 350 nm revealed a characteristic transition time of a few nanoseconds (*44*) and an incomplete collapse. In light of our observations, we conclude that the fast process reported by Wang *et al*. (*44*) is related to the formation of small clusters. For a pH-induced collapse, transition times between a few milliseconds to several seconds were reported (*39*, *43*). This suggests that the pH-induced collapse occurs on a similar time scale as the cononsolvency-induced slower (second) collapse, and hence, it is reasonable to assume that the collapse also follows the two-step process. In contrast, the collapse induced by binding glucose to phenylboronic acid groups at the polymer chains is much slower (on the scale of hundreds of seconds) because of a slow chemical reaction (*47*).

Details of the two-step process in the collapse kinetics of microgels are revealed by simulations. The fast collapse regime can be attributed to the formation and coalescence of clusters, where these clusters start to form around the cross-links after a quench, whereas the slow process is related to rearrangements of polymers and the formation of a compact globular structure. The short-time behavior observed in our simulations is different from that of linear polymers, where small clusters are formed in multiple places along the chain in the beginning of a collapse. Moreover, large voids are observed in the microgel shell during collapse, that is, there is no formation of a dense skin, which could entrap solvents inside the microgel. Hence, solvent exchange is not hampered by densification of the polymer network nor does it stimulate drainage of fluid from the inside to the outside of the microgel. Furthermore, our simulation results reveal that an increase of the relaxation time of the network polymers through an increase of the polymer length does not alter the two-stage collapse process. However, the collapse speed increases with increasing quenching depth. In addition, the collapse speed is correlated with the speed of cononsolvent transport into the microgel. This indicates that the rate-limiting factor in a temperature-induced collapse, where poor solvent conditions are instantaneous all over the entire microgel, might appear to be different from that driven by cononsolvency or change of pH. However, the two-state process persists with a less pronounced second regime (cf. fig. S19A). On the basis of simulations of linear polymers (*54*) and microgels without HIs (*64*), we conclude that the kinetics in the fast collapsing regime is driven by HIs.

In conclusion, we observed a two-step process for the microgel-to-particle collapse in our experiments and computer simulations and is a generic feature independent of the stimulus. Our results suggest that solvent diffusion and the quenching depth are the main rate-determining factors which control the volume change kinetics in microgels. Hence, the system design dictates the volume change kinetics. Together, the ability to simulate and experimentally quantify the microgel size and structural changes during the volume change process is very promising for the design of novel microgel applications.

## MATERIALS AND METHODS

### Modeling and fitting the microgel form factor during the collapse transition

The time-resolved SAXS data are highly modulated and reveal many minima and maxima over the broad *q* range. Fitting the entire scattering curve was not possible with a simple core-shell model that was used to fit experimental form factors of microgels in the equilibrium state (*36*) because a broad bump in the *q* range from 0.4 to 0.12 nm^−1^ was not fitted satisfactorily. Therefore, an additional length scale had to be introduced similarly to what was done previously for microgels with nanophase-separated internal structure (*63*). The main effect of the internal clusters is that it gives rise to a broad bump, as observed in the SAXS data. In the model, it was included as an empirical term of $e^{-R_{g}^{2}q^{2}/3}$ with a scale factor and *R*_g_ taken as a fit parameter. In the final form factor model, the density profile is defined in terms of piecewise parabola, as described by Berndt *et al*. (*36*). The density profile is based on a profile with a constant density in the center in a region up to *r* = *W* and a decay of the outer surface, which is determined by σ. The radial density profile Δρ(*r*) of a particle with such a graded surface is expressed by the half-height radius *R* = *W* + σ. Thus, the inner interface is given by *R*_in_ = *W*_core_ + σ_in_ and the outer interface by *R*_T_ = *W*_core_ + 2σ_in_ + *W*_shell_ + σ_out_. Size polydispersity of the outer radius *R*_T_ and instrumental smearing were included, the Lorentzian function *I*_L_(*q*) that described the internal polymer scattering was omitted as the *q* range, and data at high *q* did not allow for fitting with sufficient precision.

Initial model-independent analysis using a modified version of the method described by Oliveira *et al*. (*65*) for spherically symmetric particles showed that the profiles in the time-resolved series all had a low density in the core of the particles and a higher density closer to the surface. Therefore, this density distribution was used for the initial values for fitting the profiles. The final homogeneous state was first fitted on absolute scale using a contrast factor estimated from the partial density of PNIPAM in water and the calculation of the electron density of the 20 mol % of MeOH mixture. This allowed for the particle number density to be determined, and this particle number density was kept fixed in all the fits of the time-resolved series. With this information, the actual excess electron densities of the particles in the time-resolved series could be determined in units of electrons per cubic angstrom.

The model contains a high number of fit parameters. To avoid instabilities of the fits, we fixed some of the parameters in the model to reasonable values. The relative polydispersity of the size was fixed to 5%, which gave a reasonable smearing of the minima of *I*(*q*).

The surface smearing (σ_out_) was fixed to 3 nm, which is below the resolution limit of the data. We noted that usually collapsed states of microgels have sharp surfaces; hence, it was reasonable to keep the surface smearing fixed. We also experienced that the fits were not very sensitive to the electron density in the core of the particles and the width of the interface (σ_in_) between the expanded core and the collapsed outer shell. Thus, we kept the width of the interface (σ_in_) fixed at 40 nm. The core density was determined by fitting to the data at late stages of the time-resolved SAXS measurements (at about 2 s when making jumps from pure MeOH and at about 3 s when jumping from pure water), and then the core density was fixed in the fits of the time series.

**Fig. 6:**
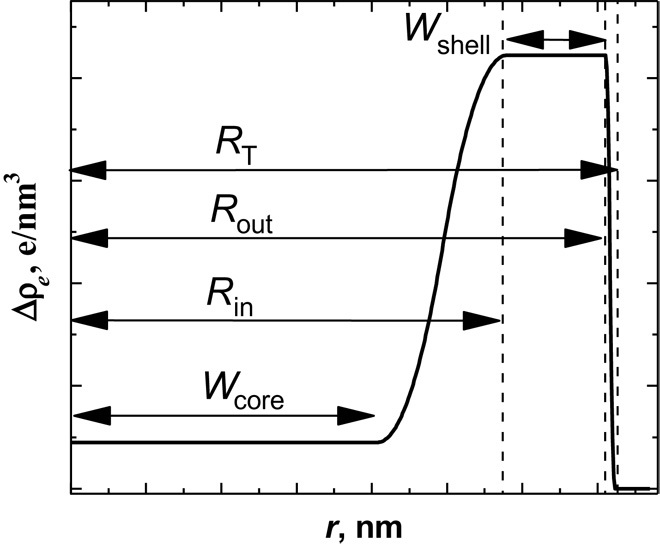
Scheme of a radial excess electron density profile for PNIPAM microgels in MeOH/H_2_O mixtures. The parameters characterizing the radial excess electron density in this form factor model are the core thickness *W*_core_, the shell thickness *W*_shell_, the inner radius *R*_in_, the outer radius *R*_out_, and the total radius *R*_T_.

In practice, an automated fitting procedure was used in which the results at a given step were used as initial values in the next step. The data were fitted in the direction from late stages to early stage. The range of the data from 0.01 to 0.12 nm^−1^ was used because of the good signal-to-noise ratio. The model was fitted to the experimental data using a least-square routine applying a reduced χ^2^ criterion. A Fourier transformation of the scattering amplitude using the electron densities instead of volume fractions of polymer gives the radial excess electron densities Δρ_*e*_ profiles in real space. We have not represented radial volume fraction profiles in real space because we have different solvents inside the microgel, which were not homogeneously distributed. Still, the polymer density profile of the microgel was closely related to the radial excess electron density (Δρ_*e*_), and it was the best representation of the radial profile that could be obtained. The radial excess electron density and the parameters describing the characteristic length scales as obtained from the form factor model are schematically shown in Fig. 6.

### Mesoscale hydrodynamic simulations

A microgel is composed of bead-spring polymers of length *N*_m_ and bead mass *M*, which are tetrafunctionally cross-linked, and is embedded in an MPC fluid. The monomers are connected via harmonic springs of equilibrium length *l*, whereas the nonbonded interactions are described by the Lennard-Jones (LJ) potential

$$U_{\text{LJ}}=\{\begin{array}{cc} 4\varepsilon [{\left(\frac{\sigma }{r_{ij}}\right)}^{12}-{\left(\frac{\sigma }{r_{ij}}\right)}^{6}]-C, & r_{ij}\leq r_{c}\\ 0, & r_{ij}>r_{c} \end{array}$$

where ε is strength (quenching depth), σ is the diameter of the monomers, *r*_*ij*_ is the distance between monomers *i* and *j*, *r*_c_ is the cutoff distance, and *C* = 4ε[(σ/*r*_c_)^12^ − (σ/*r*_c_)^6^]. The dynamics of the monomers is treated by molecular dynamics simulations, where Newton’s equations of motion are solved by the velocity-Verlet algorithm.

MPC is a particle-based mesoscale hydrodynamic simulation approach for fluids, providing a solution of Navier-Stokes equations (*49*). The MPC fluid consists of *N*_*s*_ point-like particles of mass *m*, whose dynamics proceeds in two steps: streaming and collision (*49*). During streaming, the MPC particles move ballistically. For the collision, we applied the stochastic rotation version of MPC (*49*), where the simulation box was partitioned into cubic collision cells and the MPC particle’s relative velocity, with respect to the center-of-mass velocity of all the particles in a collision cell, was rotated around a randomly oriented axis by a fixed angle. This has been shown to provide the correct hydrodynamic behavior (*51*). The coupling between the MPC fluid and the monomers is realized in the collision step, where the point-particle monomers are included in the collision (for details, see section S2.1) (*51*).

A microgel is equilibrated under good solvent conditions (that is, ε = 1.0 and *r*_c_ = 2^1/6^σ). The collapse is triggered by increasing the cutoff distance to *r*_c_ = 2.5σ with the particular quench depth. To account for cononsolvent transport, an imaginary sphere was placed around a microgel, which initially had a larger radius than a microgel. Its center coincided with the center of mass of the microgel, and its radius was reduced with a speed *v*_cosol_, whereas all the monomers outside the sphere were treated as being in the poor solvent and all monomers inside were under good solvent condition. To perform the simulations, we chose *l*, *k*_B_*T*, and *m* as units of length, energy, and mass, with the unit of time τ = (*ml*^2^/*k*_*B*_ *T*)^1/2^. The other parameters are specified in the Supplementary Materials.

## Supplementary Material

http://advances.sciencemag.org/cgi/content/full/4/4/eaao7086/DC1

## SUPPLEMENTARY MATERIALS

Supplementary material for this article is available at http://advances.sciencemag.org/cgi/content/full/4/4/eaao7086/DC1 section S1. Experimental section S2. Computer simulation fig. S1. Hydrodynamic radius as a function of the methanol mole fraction at 10° and 21°C. fig. S2. Normalized scattering curves for PNIPAM microgel at 10°C measured by SLS in the small *q* range and SAXS in the high *q* range. fig. S3. Static SAXS curves for PNIPAM microgel in *x*_MeOH_ = 0.20 at 10°C. fig. S4. Radially averaged SAXS pattern for PNIPAM in the solvent composition jump from pure MeOH to *x*_MeOH_ = 0.20 at 5 ms after the mixture. fig. S5. Radially averaged SAXS and fit curves for PNIPAM microgel in the solvent composition jump from pure MeOH to *x*_MeOH_ = 0.20. fig. S6. Radial excess electron density profiles calculated from the modeling procedure for PNIPAM microgels in the solvent composition jump from MeOH to *x*_MeOH_ = 0.20 at 10°C. fig. S7. Radially averaged SAXS patterns of PNIPAM microgels for the solvent composition change from pure H_2_O to *x*_MeOH_ = 0.20. fig. S8. Radially averaged SAXS patterns and fit curves for PNIPAM in the solvent composition jump from pure H_2_O to *x*_MeOH_ = 0.20. fig. S9. Radial excess electron density profiles calculated from the modeling procedure for PNIPAM microgels in the solvent composition jump from H_2_O to *x*_MeOH_ = 0.20 at 10°C. fig. S10. Fit results for the collapse transition of PNIPAM induced by the solvent composition jump from pure H_2_O to *x*_MeOH_ = 0.20 at 10°C. fig. S11. SAXS curves of PNIPAM in *x*_MeOH_ = 0.20 obtained by the static equilibrium measurements (squares), by the solvent composition change from MeOH (circles), and by the solvent composition change from H_2_O (triangles). fig. S12. Turbidity as a function of time for the collapse transition of PNIPAM microgel induced by changing the solvent composition from pure solvent (either H_2_O or MeOH) to *x*_MeOH_ = 0.20 at 10°C. fig. S13. Effect of the temperature on the excess enthalpy *H*^*E*^ of mixing H_2_O and MeOH. fig. S14. Schematic representation of the stopped-flow setup for the estimation of the increase of the temperature inside the TC-100/10T cuvette upon H_2_O/MeOH mixing. fig. S15. Increase of the temperature inside the TC-100/10T cuvette with time by mixing H_2_O and MeOH at 10°C to reach a final solvent composition of *x*_MeOH_ = 0.20. fig. S16. Increase of the temperature inside the TC-100/10T cuvette during the H_2_O/MeOH mixing. fig. S17. Comparison of the temperature-dependent size of PNIPAM microgel. fig. S18. Simulation results of the time evolution of $\langle R_{g}^{2}(0)\rangle -\langle R_{g}^{2}(t)\rangle$ for microgels with different quenching depths ε and different polymer lengths *N*_m_. fig. S19. Results from simulations for evolution of the microgel size and collapse velocity. fig. S20. Results from simulations for monomer distribution and microgel conformations. table S1. Fit results for PNIPAM microgel in pure H_2_O, pure MeOH, and *x*_MeOH_ = 0.20 at 10°C. movie S1. Animation of microgel conformational changes during collapse for ε = 2.5. References (*66*–*74*)
