# Supplementary material for: Time-resolved structural evolution during the collapse of responsive hydrogels: The microgel-to-particle transition
Source: Sci Adv. 2018 Apr 6;4(4):eaao7086. doi: 10.1126/sciadv.aao7086 (PMC5938240; doi:10.1126/sciadv.aao7086)
Supplement: http://advances.sciencemag.org/cgi/content/full/4/4/eaao7086/DC1 [file supp_4_4_eaao7086__index.html]

Science Advances | Science Advances

## Supplementary Materials

**This PDF file includes:**

- section S1. Experimental
- section S2. Computer simulation
- fig. S1. Hydrodynamic radius as a function of the methanol mole fraction at 10° and 21°C.
- fig. S2. Normalized scattering curves for PNIPAM microgel at 10°C measured by SLS in the small *q* range and SAXS in the high *q* range.
- fig. S3. Static SAXS curves for PNIPAM microgel in *x*MeOH = 0.20 at 10°C.
- fig. S4. Radially averaged SAXS pattern for PNIPAM in the solvent composition jump from pure MeOH to *x*MeOH = 0.20 at 5 ms after the mixture.
- fig. S5. Radially averaged SAXS and fit curves for PNIPAM microgel in the solvent composition jump from pure MeOH to *x*MeOH = 0.20.
- fig. S6. Radial excess electron density profiles calculated from the modeling procedure for PNIPAM microgels in the solvent composition jump from MeOH to *x*MeOH = 0.20 at 10°C.
- fig. S7. Radially averaged SAXS patterns of PNIPAM microgels for the solvent composition change from pure H2O to *x*MeOH = 0.20.
- fig. S8. Radially averaged SAXS patterns and fit curves for PNIPAM in the solvent composition jump from pure H2O to *x*MeOH = 0.20.
- fig. S9. Radial excess electron density profiles calculated from the modeling procedure for PNIPAM microgels in the solvent composition jump from H2O to *x*MeOH = 0.20 at 10°C.
- fig. S10. Fit results for the collapse transition of PNIPAM induced by the solvent composition jump from pure H2O to *x*MeOH = 0.20 at 10°C.
- fig. S11. SAXS curves of PNIPAM in *x*MeOH = 0.20 obtained by the static equilibrium measurements (squares), by the solvent composition change from MeOH (circles), and by the solvent composition change from H2O (triangles).
- fig. S12. Turbidity as a function of time for the collapse transition of PNIPAM microgel induced by changing the solvent composition from pure solvent (either H2O or MeOH) to *x*MeOH = 0.20 at 10°C.
- fig. S13. Effect of the temperature on the excess enthalpy *H*E of mixing H2O and MeOH.
- fig. S14. Schematic representation of the stopped-flow setup for the estimation of the increase of the temperature inside the TC-100/10T cuvette upon H2O/MeOH mixing.
- fig. S15. Increase of the temperature inside the TC-100/10T cuvette with time by mixing H2O and MeOH at 10°C to reach a final solvent composition of *x*MeOH = 0.20.
- fig. S16. Increase of the temperature inside the TC-100/10T cuvette during the H2O/MeOH mixing.
- fig. S17. Comparison of the temperature-dependent size of PNIPAM microgel.
- fig. S18. Simulation results of the time evolution of ‹R2g (0) › − ‹R2g (t) › for microgels with different quenching depths ε and different polymer lengths *N*m.
- fig. S19. Results from simulations for evolution of the microgel size and collapse velocity.
- fig. S20. Results from simulations for monomer distribution and microgel conformations.
- table S1. Fit results for PNIPAM microgel in pure H2O, pure MeOH, and *x*MeOH = 0.20 at 10°C.
- References (*66–74*)

Download PDF

**Other Supplementary Material for this manuscript includes the following:**

- movie S1 (Microsoft Excel format)

**Files in this Data Supplement:**

- Adobe PDF - aao7086\_SM.pdf
