# Supplementary material for: Time-resolved structural evolution during the collapse of responsive hydrogels: The microgel-to-particle transition
Source: Sci Adv. 2018 Apr 6;4(4):eaao7086. doi: 10.1126/sciadv.aao7086 (PMC5938240; doi:10.1126/sciadv.aao7086)
Supplement: http://advances.sciencemag.org/cgi/content/full/4/4/eaao7086/DC1 [file aao7086_SM.pdf]

Science Advances

AAAS

advances.sciencemag.org/cgi/content/full/4/4/eaao7086/DC1

Supplementary Materials for

Time-resolved structural evolution during the collapse of responsive hydrogels: The microgel-to-particle transition

Rico Keidel, Ali Ghavami, Dersy M. Lugo, Gudrun Lotze, Otto Virtanen, Peter Beumers, Jan Skov Pedersen, Andre Bardow, Roland G. Winkler, Walter Richtering

Published 6 April 2018, *Sci. Adv.* **4**, eaao7086 (2018)
DOI: 10.1126/sciadv.aao7086

The PDF file includes:

- section S1. Experimental
- section S2. Computer simulation
- fig. S1. Hydrodynamic radius as a function of the methanol mole fraction at 10° and 21°C.
- fig. S2. Normalized scattering curves for PNIPAM microgel at 10°C measured by SLS in the small q range and SAXS in the high q range.
- fig. S3. Static SAXS curves for PNIPAM microgel in x_{MeOH} = 0.20 at 10°C.
- fig. S4. Radially averaged SAXS pattern for PNIPAM in the solvent composition jump from pure MeOH to x_{MeOH} = 0.20 at 5 ms after the mixture.
- fig. S5. Radially averaged SAXS and fit curves for PNIPAM microgel in the solvent composition jump from pure MeOH to x_{MeOH} = 0.20.
- fig. S6. Radial excess electron density profiles calculated from the modeling procedure for PNIPAM microgels in the solvent composition jump from MeOH to x_{MeOH} = 0.20 at 10°C.
- fig. S7. Radially averaged SAXS patterns of PNIPAM microgels for the solvent composition change from pure H_2O to x_{MeOH} = 0.20.
- fig. S8. Radially averaged SAXS patterns and fit curves for PNIPAM in the solvent composition jump from pure H_2O to x_{MeOH} = 0.20.
- fig. S9. Radial excess electron density profiles calculated from the modeling procedure for PNIPAM microgels in the solvent composition jump from H2O to xMeOH = 0.20 at 10°C.
- fig. S10. Fit results for the collapse transition of PNIPAM induced by the solvent composition jump from pure H_2O to x_{MeOH} = 0.20 at 10°C.

- fig. S11. SAXS curves of PNIPAM in x_{MeOH} = 0.20 obtained by the static equilibrium measurements (squares), by the solvent composition change from MeOH (circles), and by the solvent composition change from H_2O (triangles).
- fig. S12. Turbidity as a function of time for the collapse transition of PNIPAM microgel induced by changing the solvent composition from pure solvent (either H_2O or MeOH) to x_{MeOH} = 0.20 at 10°C.
- fig. S13. Effect of the temperature on the excess enthalpy H^E of mixing H_2O and MeOH.
- fig. S14. Schematic representation of the stopped-flow setup for the estimation of the increase of the temperature inside the TC-100/10T cuvette upon H_2O/MeOH mixing.
- fig. S15. Increase of the temperature inside the TC-100/10T cuvette with time by mixing H2O and MeOH at 10°C to reach a final solvent composition of x_{MeOH} = 0.20.
- fig. S16. Increase of the temperature inside the TC-100/10T cuvette during the H_2O/MeOH mixing.
- fig. S17. Comparison of the temperature-dependent size of PNIPAM microgel.
- fig. S18. Simulation results of the time evolution of $\left\langle R_g^2(0)\right\rangle - \left\langle R_g^2(t)\right\rangle$ for microgels with different quenching depths ε and different polymer lengths N_m.
- fig. S19. Results from simulations for evolution of the microgel size and collapse velocity.
- fig. S20. Results from simulations for monomer distribution and microgel conformations.
- table S1. Fit results for PNIPAM microgel in pure H_2O, pure MeOH, and x_{MeOH} = 0.20 at 10°C.
- References (*66–74*)

Other Supplementary Material for this manuscript includes the following:
(available at advances.sciencemag.org/cgi/content/full/4/4/eaao7086/DC1)

- movie S1 (.mov format). Animation of microgel conformational changes during collapse for ε = 2.5.

SUPPLEMENTARY MATERIALS

section S1. Experimental

section S1.1. Materials

N-isopropylacrylamide (NIPAM: Acros Organic, > 99 %), initiator potassium peroxide sulfate (KPS: Merck, 99 %), crosslinker N,N'-methylenebisacrylamide (BIS: Alfa Aesar, > 99.5 %) and Methanol (MeOH: VWR, 99.5 %) were used without further purification. Doubly distilled Milli-Q water was used during the synthesis and for the preparation of the samples.

section S1.2. Microgel Synthesis

The microgel was synthesized via temperature-ramp, surfactant-free free-radical precipitation-polymerization following the routine proposed by Lyon et al. *(66)* as this allows preparing larger microgels required for the present study to make the time scales fit into the experimentally accessible window. This does not affect the general structure of the microgel as discussed further below. NIPAM (32 mmol) and BIS (0.08 mmol) were dissolved in 230 mL of doubly distilled Milli-Q water. After dissolution (30 minutes), the monomer solution was filtered into a 1000 mL three-necked reaction vessel through glass wool. Doubly distilled Milli-Q water (15 ml) was used to wash the glass wool throughout filtration. The reaction vessel was equipped with a stirrer, reflux condenser, IR thermometer, nitrogen (N_2) inlet and injection inlet. CEM Mars 5 Microwave Accelerated Reaction System was programmed to heat NIPAM-BIS mixture from room temperature to initial synthesis temperature (T_i = 45 °C) in two minutes (400 W) and hold it at a stir rate of 400 rpm for 1 h while purging the reaction system with N_2. Afterwards, 5 mL aliquot of 0.155 M of degassed aqueous solution of the initiator KPS was injected to the reaction vessel. The temperature was kept at T_i (45 °C) for a nucleation time (t_{nucl}) of 30 min and then ramped to T_f = 65 °C at a ramp rate of 0.66 K/min. Upon reaching 65 °C, the reaction proceeded for 3 h. Immediately after the polymerization time, the reaction mixture was filtered through glass

wool to remove small amount of large aggregates. The microgel was purified by three subsequent centrifugations (1 h at 50 000 rpm) and the supernatant was redispersed in doubly distilled Milli-Q water. The purified product was freeze-dried for 48-72 h.

section S1.3. Static Light Scattering (SLS)

The scattering intensity was measured at scattering angles from 15° to 145° and at 10 and 20 °C using a modified SOFICA goniometer (SLS-Systemtechnik GmbH) equipped with laser light sources of 633 nm and 405 nm, respectively.

section S1.4. Dynamic Light Scattering (DLS)

The thermo-responsive behavior of the PNIPAM microgels at different H_2O/MeOH compositions was studied by means of DLS at a scattering angle of 30° and wave length of 633 nm. Figure S1 depicts the hydrodynamic radius (computed via the Stokes-Einstein equation using the proper value of the solvent viscosity) of the microgel at different solvent compositions at 10 and 21 °C. The microgel is swollen in the pure solvents and a minimum size is reached at $x_{\mathrm{MeOH}} = 0.2$.

section S1.5. SAXS & SLS measurement in equilibrium states

Figure S2 shows the normalized scattering curves obtained by SLS and SAXS for microgel solutions in the initial swollen state (i.e. in pure H_2O and in pure MeOH at 10 °C) as well as in the collapsed state at $x_{\mathrm{MeOH}} = 0.20$. The solid lines display the fit to the experimental data obtained by the fuzzy spheres model described by Stieger et. al. *(37).*

The oscillations are correctly reproducing the data, but there are some additional effects concerning the depth and height of minima and maxima that are not accounted for. We speculate that with the extended q-range we are more sensitive to the resolution function, polydispersity and possible deviations from spherical symmetry. However, these influences are not important

because the TR-SAXS data can be fitted without including additional effects. The fit results are summarized in table S1.

The scattered intensities of collapsed PNIPAM in x_{MeOH} = 0.20 obtained from static measurements at the beamline ID02 are displayed in fig. S3. The solid line is the fit to the experimental data obtained by the fuzzy sphere model. In table S1 summarizes the fit results.

The scattering curve exhibit several form factor minima, which are shifted to higher q values in comparison to the position of the minima of PNIPAM in pure H_2O or MeOH fig. S2, indicating particles of smaller sizes and with a narrow size distribution. The scattering intensity decays with q^{-4} in the high q regime indicating a sharp particle surface similar to compact spheres.

The fuzziness of the particle surface described by σ_{surf} is very small for PNIPAM in x_{MeOH} = 0.20 (table S1) indicating that the surface chains are collapsed and shows that the microgel has the structure of a sphere with homogeneous density profile. This small σ_{surf} value is in good agreement with those reported by Stieger et al. *(37)*, when PNIPAM microgel in H_2O are in the shrunken state at high temperatures (39 °C). On the other hand, σ_{surf} values of PNIPAM dispersed in pure solvents are larger due to the fuzzy character of swollen microgels. For better characterization (see Stieger et al. *(37)*) R is defined here as the radius with half of the excess electron density compared to the core. The total microgel radius R_T in x_{MeOH} = 0.20 is less than half of its size in the pure solvents.

section S1.6. Stopped-flow experiments with TR-SAXS

SAXS experiments were collected at the High Brilliance beamline “ID02 - Time-Resolved Ultra Small-Angle X-Ray Scattering” at the European Synchrotron Radiation Facility (ESRF) in Grenoble, France, using 12.4 keV photons (corresponding to a wavelength of λ = 0.1 nm). The X-ray scattering intensity was recorded using a FReLoN (Fast-Readout Low-Noise) Kodak CCD

detector. A detector binning of 2 x 2 was used. The sample-detector distance was set to 8 m covering a q-range of approximately 0.01 - 0.2 nm^{-1}.

Static SAXS measurements were performed in a flow-through capillary cell of 1.88 mm diameter to obtain the scattering patterns of the initial and final state of our microgels at 10 °C. The samples were thermally stabilized at 10 °C. The scattering patterns obtained for the microgels at the initial and final state as well the background solutions (H_2O, MeOH and x_{MeOH} = 0.20) were averaged over 20 frames to improve statistics.

Time-resolved scattering patterns were collected using a Bio-Logic SFM-400 stopped-flow device with four syringes *(48)*. Samples were stored in a fridge at a temperature of ~ 8 °C. The microgel solutions in pure solvent (either H_2O or MeOH) were injected from syringe 4, the cononsolvent from the third one and water from the first and second syringes to clean the stopped-flow cell between shots and to calibrate the scattering intensity to absolute scale. The reservoir for the four syringes and stopped-flow cell were kept at 10 °C by circulating water around the reservoir and observation chamber. The observation chamber consisted of the stopped-flow cell (quartz capillary with wall thickness of approximately 10 μm and diameter 1.35 mm) enclosed in an aluminium holder. The volume mixing ratios were calculated based on the initial and the final solvent composition of the PNIPAM dispersion. The final concentration of the PNIPAM dispersion in x_{MeOH} = 0.20 was set to 0.40 wt%. The stopped-flow cell was homogeneously filled by injecting a total volume of ~ 350 μL of the mixed solution. The total flow rates for the jump from pure water and pure methanol were 7.04 mL s^{-1} and 8.54 mL s^{-1} respectively. PNIPAM dispersion in pure solvent was mixed turbulently with the cononsolvent and injected for at least 50 ms continuously into the flow path of the stopped-flow device corresponding to a steady state condition. X-ray data acquisition was triggered directly at 50 ms and later according to the time steps. The sample age of the mixed solution during this steady state was mainly determined by the necessary time to flow from the mixer inside the stopped-flow

device to the point of X-ray exposure. The transfer time from the last mixer to the capillary cell was estimated to be approximately 3.00 ms. The effective exposure time for each frame was 1.5 ms, i.e., each scattering curve was integrated over 1.5 ms. Consequently, the first scattering curve describes the kinetics at a time of 5 ms. The minimum detector readout time during the experiment was 320 ms (2 x 2 binning), hence a stroboscopic data strategy was applied to access points in time well before 320 ms. This was achieved by varying the dead time before the first X-ray exposure, i.e. a delay time of 40 ms, 50 ms, 55 ms, 60 ms, 70 ms etc. was introduced. Each acquisition was repeated at least three times to improve the statistics and to verify the reproducibility. Subsequently, all curves were averaged for each point in time.

The time axis for the measurement data was calculated with the following equation

$$\max(\tau_{SF},\ t_{onset}+t_{exp}/2+\tau_{SF}-t_{mix}), \tag{S1}$$

where τ_{SF} is the dead time of the stopped-flow device, t_{onset} is the onset time of the frame, t_{exp} the exposure time or lifetime of the beam and t_{mix} the mixing time for the purge to reach steady state.

The SAXS data was reduced (i.e., normalized, regrouped into one-dimensional patterns, averaged, and background-subtracted) using ESRF's SAXS utilities software *(67)*.

Measurements of the size of the microgels before and after passing the mixer showed no difference, thus the microgels are not destroyed when passing the mixer.

section S1.7. Form factor model comparison

For the analysis of the diffraction patterns obtained through time-resolved SAXS (TR-SAXS), a form factor model is necessary. There are various form factor models which differ in complexity available in literature. For microgels with a constant excess electron density profile which smoothly drops at the edge *(36)*, a fuzzy sphere model is applied. For microgels with a more complex density profile a core-shell fuzzy sphere model *(37)* is used. This core-shell fuzzy sphere

model allows for two sections with different excess electron densities and smooth transitions. For the fit of our TR-SAXS measurement data we tried the models of different complexity for the different states during the microgel collapse. Figure S4 shows a comparison of the form factor models fitted to the TR-SAXS measurement at 5 ms.

section S1.8. Modelling of the Scattering Curves

Scattering data are an intensity distribution $I(q)$ as a function of momentum transfer $q = (4\pi/\lambda)\sin\theta$, where 2θ is the scattering angle. For data on absolute scale, it is usual to express the scattering intensity distribution in terms of the differential scattering cross section $\frac{d\sigma(q)}{d\Omega}$. It can for a very dilute dispersion of monodisperse and spherical particles without concentration effects be expressed as

$$\frac{d\sigma(q)}{d\Omega} = n\Delta\rho_{SAXS}^{2} V_{pol}^{2} P(q) \quad \text{(S2)}$$

where n is the number density of microgel particles, $\Delta\rho_{SAXS}$ is the excess scattering length density between polymer and solvent, V_{pol} is the 'dry' volume of the polymer in a particle and $P(q)$ is the normalized scattering form factor ($P(q=0) = 1$) of the particle.

The number density of microgel particles n can be calculated from the mass fraction of microgel in the sample c as follows

$$n=c/(V_{pol}\rho_{polymer}^{d})/(\frac{c}{\rho_{polymer}^{d}} + \frac{[1-c]}{\rho_{solvent}}) \quad \text{(S3)}$$

where $\rho_{polymer}^{d}$ is apparent specific density of the polymer and ρ_{solvent} is the density of the solvent.

For SAXS the excess scattering length density is proportional to the electron density difference of the polymer $\rho_{e,polymer}$ and of the solvent $\rho_{e,solvent}$

$$\Delta\rho_{SAXS} = b_e\left(\rho_{e,polymer} - \rho_{e,solvent}\right) \quad \text{(S4)}$$

where b_e is the electron scattering length (2.82×10^{-6} nm, the classical Thomson radius).

The electron density (electrons per unit of volume) of the polymer can be calculated from its apparent specific density $\rho^d_{polymer}$ as follows

$$\rho_{e,polymer} = \rho^d_{polymer} N_A Z_{polymer}/M_{polymer} \quad \text{(S5)}$$

and similarly for the solvent

$$\rho_{e,solvent} = \rho_{solvent} N_A \left[\frac{\varphi_{MeOH} Z_{MeOH}}{M_{MeOH}} + \frac{(1-\varphi_{MeOH}) Z_{H2O}}{M_{H2O}}\right] \quad \text{(S6)}$$

where Z_i and M_i denote, respectively, the number of electrons and molecular weight of species i, N_A is Avogadro's number, and φ_{MeOH} is the weight fraction of methanol in the solvent.

The size polydispersity of particle has been considered in the modelling and fitting of the data. It is described by a normalized Gaussian number distribution as

$$D(R, \langle R\rangle, \sigma_{poly}) = \frac{1}{\sqrt{2\pi\sigma^2_{poly}\langle R\rangle^2}} exp\left(-\frac{(R-\langle R\rangle)^2}{2\sigma^2_{poly}\langle R\rangle^2}\right) \quad \text{(S7)}$$

with $\langle R\rangle$ describing the average particle radius and σ_{poly} denoting the relative particle size polydispersity.

Thus, the differential scattering cross section is expressed as

$$\frac{d\sigma(q)}{d\Omega} = n\Delta\rho^2_{SAXS} \int_0^\infty D(R, \langle R\rangle, \sigma_{poly}) V_{pol}(R)^2 P(q,R)\, dR \quad \text{(S8)}$$

For the SAXS data of the large particle investigated in the present work, there are significantly instrumental smearing effects and these have to be taken into account. It is done by introducing a resolution function $R(\langle q\rangle, q)$ which describes the probability distribution of the actual scattering vectors q for a given nominal scattering vector $\langle q\rangle$. The parameters of the resolution function can be estimated from the geometry of the setup, the wavelength distribution and the detector

resolution. The instrument resolution for the beamline ID02 is approximately described by a Gaussian function as follows

$$R(\langle q\rangle, q) = \frac{1}{\sigma\sqrt{2\pi}} \exp\left(-(q - \langle q\rangle)^2/\sigma^2\right) \tag{S9}$$

where the width of the instrument smearing is σ=0.0015nm^{-1}.

The model intensity is thus described by

$$I^{\text{mod}}(\langle q\rangle) = \int_0^\infty \frac{d\sigma(q)}{d\Omega} R(\langle q\rangle, q)\, dq \tag{S10}$$

Different form factor functions $P(q,R)$ have been used to fit the scattering curves, depending on in which state is the PNIPAM microgel, as will be described below.

section 1.9. Modelling and fitting of the microgel form factor in the initial and final states

Scattering data for PNIPAM microgels have been successfully described by a fuzzy sphere model *(37)*. The smooth decay of the density profile of the microgel at its periphery arises from an inhomogeneous distribution of cross-linker within the particle. This is modeled in real space as a convolution of the radial scattering length distribution of a compact sphere with a Gaussian to get a function with a gradual drop-off in scattering length density generating the fuzziness of the particle surface, described by the form factor $P(q)$

$$P(q) = [A(q)]^2 \qquad \text{with}$$

$$A(q) = \left(\frac{3[\sin(qR) - qR\cos(qR)]}{(qR)^3} \times \exp\left(-\frac{(\sigma_{surf}q)^2}{2}\right)\right) \tag{S11}$$

where $A(q)$ defines the amplitude of the form factor $P(q)$. R and σ_{surf} are the two adjustable parameters, representing the radius of the particle where the scattering length density profile decreased to half the core density and the width of the smeared particle surface, respectively. The core of the microgel that exhibit a higher degree of cross-linking density is described by the radial

box profile extending to a radius of about $R_{box} = R - 2\sigma_{surf}$. In dilute solution, the profile approaches zero at $R_T = R + 2\sigma_{surf}$. Thus, the overall size of the particle is approximated given by R_T. A small number of chains reaching outside the particle will contribute only to the hydrodynamics of the particle, and therefore the size obtained by scattering methods is, expected to be smaller than the hydrodynamic radius R_H determined by DLS.

To account for polymer-like scattering due internal structure of the microgel layer, a Lorentzian function $I_L(q) = I_L(0)/[1+q^2\xi^2]$ is added to $P(q)$ in Eq. S11. The average correlation length in the network is described by ξ and $I_L(0)$ is the value of the intensity arising from fluctuations for $q \to 0$. Finally, a constant background I_{back} is added to correct the residual incoherent scattering.

Incorporating all above-mentioned contributions, the model expression for the scattering intensity distribution is given by

$$I^{mod}(q) = n\Delta\rho_{SAXS}^2 \int_0^\infty \int_0^\infty R(\langle q\rangle, q) D\left(R, \langle R\rangle, \sigma_{poly}\right) \times V_{pol}(R)^2 [A(q)^2 + I_L(q) + I_{back}] dR dq$$

(S12)

section 1.10. Modelling and fitting the microgel form factor during the collapse transition

More complex structures as core-shell microgel for the form factor model have been used to describe the time-resolved series of SAXS curves measured for both solvent composition jumps investigated in the present contribution. See fig. S5, S7 and S8.

As described above, the time-resolved series data have a bump at higher q, which was impossible to fit with the simple core-shell model proposed by Berndt, Pedersen and Richtering *(36)*. We ascribe the bump to internal density fluctuations as one has in the core-shell fuzzy sphere model with density fluctuations *(63)*. An empirical term of $e^{-R_g^2 q^2/3}$ was included with a scale factor and R_g taken as a fit parameter.

In this model, the density profile is defined in terms of piecewise parabola as is described in Ref. *(36)*. The density profile is based on a profile with a constant density in the center in a region up to $r = W$ and a decay of the outer surface, which is determined by σ. The radial density profile $\Delta\rho$(r) of a particle with such a graded surface is expressed by the half-height radius $R = W + \sigma$. Thus, the inner interface is given by $R_{in} = W_{core} + \sigma_{in}$; and the outer interface by $R_T = W_{core} + 2\sigma_{in} + W_{shell} + \sigma_{out}$.

Size polydispersity of the outer radius R_T (Eq. S7), and instrumental smearing (Eq. S9) were included, however the Lorentzian function $I_L(q)$ that described the internal polymer scattering was omitted as the q-range and data at high q do not allow inclusion of this term.

An initial model-independent analysis using an approach similar to that described in *(65)* showed that the profiles in the time-resolved series all have a low density in the core of the particles and a higher density closer to the surface. Therefore, this was the initial guess for the profiles.

The final homogeneous state was first fitted on absolute scale using a contrast factor estimated from the partial density of PNIPAM in water and calculation of the electron density of the 20 mol % of MeOH mixture. This allowed the particle number density to be determined and this was kept fixed in all the fits of the time-resolved series. With this, the actual excess electron densities of the particles in the time-resolved series could be determined in units of electrons per cubic Angstrom.

The model contains a high number of fit parameters and this gave some instabilities of the fits. Therefore, it was decided to fix some of the parameters in the model to reasonable values. Precipitation polymerization of NIPAM leads to microgels with very narrow size distribution (<10 %) and fixing the relative polydispersity of the size to 5 % give a reasonable smearing of the minima in the intensity expression as a function of q and it is also in agreement with the value determined by dynamic light scattering. The surface smearing (σ_{out}) was fixed to 3 nm, which is below the resolution limit of the data. We note that usually collapsed states of microgels have sharp surfaces so therefore it is reasonable to keep it fixed at a low value *(37)*. We also

experienced that the fits were not very sensitive to the electron density in the core of the particles and the width of the interface (σ_{in}) between the expanded core and the collapsed outer shell. Therefore, we kept the width of the interface (σ_{in}) fixed at 40 nm. And the core density was determined by fitting to the data at late stages of the time-resolved measured (at about 2 s when making jumps from pure MeOH and at about 3 s when jumping from pure water) and fixed in the fits of the time series.

In practice, an automated fitting procedure was used in which the results at a given step is used as initial values in the next step. The data were fitted in the direction from late stages to early stage. The range of the data from 0.01 to 0.12 nm^{-1} was used, which seems to have good signal to noise ratio.

The model was fitted to the experimental data using a least-square routine minimizing the reduced χ^2 criterion. A Fourier transformation of the scattering amplitude using the electron densities instead of volume fractions of polymer gives the radial excess electron densities $\Delta\rho_e$ profiles in real space. We have not represented radial volume fraction profiles in real space as is illustrated in Stieger et. al. *(37)*, because we have different solvents inside the microgel, which are not homogeneously distributed, rendering the usual approach impossible.

section S1.11. Additional electron density profiles

Figure S6 shows further radial excess electron density $\Delta\rho(r)$ profiles, obtained from the modelling procedure, for the solvent composition change from MeOH to $x_{MeOH} = 0.2$. The analogous plot for the solvent composition change starting from water to $x_{MeoH} = 0.2$ is presented in fig. S9.

section S1.12. Comparison of measurements with the stopped-flow device after 2000 ms and of the static SAXS measurement at $x_{\mathbf{MeOH}}$ = 0.20

Figure S11 shows data obtained (i) with the stopped-flow device at long times and (ii) in a static SAXS experiment. The data overlap in the low-*q* region and the position of the first minimum is identical. This indicates that the microgels have reached the final size in the time-resolved experiment. However, the curves are different in the high-*q* regions. This indicates that solvent composition inside the microgel has not yet reached the equilibrium value. Please note that the phase diagram shows a rather broad minimum, where the size does not depend strongly on solvent composition (see fig. S1), however the electron density differs.

section S1.13. Time-resolved turbidity

Transition kinetics of PNIPAM were also investigated via change of turbidity using a Bio-Logic SFM-3000/S stopped-flow device with three syringes and two mixers equipped with a MPS 70 microprocessor and a spectrometer MOS-200. The light source was a Xe lamp with shielded lamp housing at 150 W adjusted to a wavelength of 500 nm. The detector was a photomultiplier tube connected to the observation chamber at 180° with respect to the light source. A Berger ball mixer and a TC-100/10T cuvette were used. The TC-100/10T cuvette was made from black and transparent quartz (Suprasil 2 grade B), with a wall thickness of 300 μm and a light path of 10 mm with an aperture of 1.0 mm. The temperature was set to 10 °C to be far away from the VPT when the temperature rises due to the heat of mixing (see section 1.15). The sample temperature was maintained at the set temperature by circulating water around the reservoir and observation chamber. The samples were kept in the reservoir ca. 1 h before the measurements. The data acquisition was set to start 10 ms (pre-trigger time, $t_{\text{pre-trigger}}$) before the flow stops. Time resolution was 1 ms.

PNIPAM microgels dissolved in pure solvent (either H_2O or MeOH), H_2O, and MeOH were injected into the stopped-flow apparatus. PNIPAM solution and its cononsolvent were mixed and the final concentration of the PNIPAM in x_{MeOH} = 0.20 was set to 0.40 wt%. A total sample volume of ~ 330 μL and a total flow rate of 5.50 mL/s were used. Under these conditions, the dead time (t_{dead}) of the instrument was 5.50 ms.

The mixing ratio was calculated based on the initial and the final solvent composition. Each kinetics measurement corresponds to an average of 5-10 runs to improve statistics and to ensure reproducibility. For all measurements, the times were corrected for t_{dead} and the $t_{pre\text{-}trigger}$.

The turbidity τ is defined as *(68)*

$$\tau = l^{-1} \ln\left(\frac{I_0}{I_t}\right) \tag{S13}$$

where I_0 is the intensity of the incident light, I_t the transmitted intensity and l the length of the optical path.

TR-turbidity of collapse transition kinetics of PNIPAM microgels

Figure S12 displays the evolution of turbidity of the microgel collapse induced by solvent exchange. The turbidity increases immediately after mixing with the other solvent. The increase of turbidity is due to the relative higher refractive index difference of the collapsed polymer network. The turbidity reaches the same plateau value in both cases as the same final state is reached.

The change of turbidity can be fitted well with a two exponential function suggesting that two processes can be observed. The characteristic times of this process τ_1 and τ_2 are estimated by fitting the experimental data. The switching times obtained for starting from pure H_2O are τ_1= ~

2.1 ms and τ_2= ~ 171 ms respectively (from pure MeOH the switching times are τ_1= ~ 1.8 ms and τ_2= ~ 128 ms respectively).

section S1.14. Thermodynamic properties of H2O/MeOH mixtures

Thermodynamic properties of H_2O/MeOH mixtures show an anomalous behavior *(69,70).* The excess enthalpy of mixing (H^E) H_2O and MeOH is negative. It reaches a maximum negative value at ca. x_{MeOH} = 0.20 – 0.40, depending on the temperature of the system. In fig. S13 a comparison of the experimental data (dots) taken from ref. *(70)* is displayed with theoretical calculations (solid lines) obtained from a non-random two-liquid model (NRTL) *(71).* As it can be observed, the experimental data and the theoretical calculations agree very well.

The strongly negative enthalpy of mixing H_2O and MeOH inside the stopped-flow cell can cause an increase of the temperature. Since PNIPAM microgels are thermosensitive particles, it is crucial to measure and to control the temperature inside the stopped flow cuvette upon phase transition induced by H_2O/MeOH mixing.

The increase of temperature inside the SF cuvette upon H_2O and MeOH mixing was measured experimentally (as will be explained in detailed below) and estimated numerically using Eq. (S14) for the heat capacity C_p definition *(70)*

$$\Delta T = -\frac{H^E}{C_p} \qquad \text{(S14)}$$

with heat capacity C_p in J/mol K and excess enthalpy mixing H^E in J/mol.

section S1.15. Evaluation of the temperature increase inside the stopped-flow cuvette for the turbidity experiment

The increase of the temperature inside the TC-100/10T cuvette before the measurement and during the mixing of MeOH and H_2O was performed by using a cable sensor Pt100 installed to a digital thermometer (fig. S14). The change in temperature with time was recorded using ESM-4450 software (from National Instruments Labview^{TM}) installed to the PC of the stopped-flow device.

The temperature in the thermostat was set to 10 °C. The reservoir chambers for syringe 2 and 3 were filled with MeOH and H_2O, respectively. Both liquids were let there for 30 min in order to ensure that they have reached the temperature of 10 °C. Afterwards, the liquids were pushed up from the reservoirs to the mixer and to the cuvette. The mixture flowed through the cuvette for more than 40 s.

As displayed in fig. S14 and fig. S16 the temperature inside the cuvette was around 11.5 °C at the time t = 0 s (before mixing). This means the temperature was 1.5 °C higher inside the cuvette than set in the water bath of the thermostat coupled to the stopped-flow device (temperature 10 °C). When the liquids were pushed up from the syringes to the mixer and to the cuvette, the temperature increased until a maximum temperature was reached and was kept at this value for a few seconds, before it decreased with a slower rate to the initial value. The increase in temperature inside the cuvette was around 11.3 K, when H_2O and MeOH were mixed to get a final solvent composition of 20 mol % MeOH, which is very close to the theoretical predictions that we have made by assuming an adiabatic process without friction. The same procedure was performed for different H_2O/MeOH mixtures. The resulting temperature changes inside the cuvette are summarized in fig. S15.

section S1.16. Temperature dependent size of the PNIPAM microgel as determined by DLS

As mentioned in the previous chapter the enthalpy of mixing can lead to a temperature rise, which could have an effect on the microgel collapse transition. Therefore, we adapted the starting temperature of the system, such that an increase of the temperature inside the stopped-flow cell will not affect significantly the volume change of the microgel. Figure S17 displays the size of the microgel (hydrodynamic radius) as function of temperature in the pure solvents and at x_{MeOH} = 0.2. The figure shows a temperature increase of ca. 10 K which does not affect the initial size and hardly the final state, thus we can conclude that the microgel collapse is mainly determined by the change of the solvent composition.

section S2. Computer simulation

We apply a hybrid simulation approach, combining Molecular Dynamics Simulations (MD) for microgel particles with Multiparticle Collision Dynamics (MPC) simulations for the embedding fluid.

section 2.1. Multiparticle collision dynamics approach

MPC is a mesoscale hydrodynamic simulation approach for fluids *(49,72,73)*. Thereby, the fluid is presented by N_s point particles of mass m distributed in a cubic simulation box of length L with periodic boundary conditions. The dynamics of the fluid particles proceeds by subsequent streaming a collision steps. In the streaming step, MPC particles move ballistically within the time interval h

$$\boldsymbol{r}_i(t+h) = \boldsymbol{r}_i(t) + h\boldsymbol{v}_i(t) \tag{S15}$$

where $\boldsymbol{r}_i$ and $\boldsymbol{v}_i$ are the position and velocity of particle i. In the collision step, the solvent particles are sorted in cubic collision cells of length a. Subsequently, the relative velocity of a

particle, with respect to the center-of-mass velocity of the cell $\boldsymbol{v}_{cm}$, is rotated by a fixed angle α around a randomly oriented axis, i.e.

$$\boldsymbol{v}_i(t+h) = \boldsymbol{v}_{cm}(t) + \mathbf{R}(\alpha)(\boldsymbol{v}_i(t) - \boldsymbol{v}_{cm}(t)) \tag{S16}$$

where $\mathbf{R}(\alpha)$ is the rotation matrix. For each collision step, a random shift of the entire collision grid is applied in order to ensure Galilean invariance of the system *(74)*.

section 2.2. Molecular dynamics simulations: Microgel model

Microgel particles are represented as a regular network of polymers of length N_m, which are tetrafunctionally crosslinked. The monomers are modeled as pointlike particles, each of mass M. They are connected through the harmonic potential

$$U_{i,i+1}^{b} = \frac{K}{2}(|\boldsymbol{r}_{i+1} - \boldsymbol{r}_i| - l \tag{S17}$$

where K is the spring constant and l is the equilibrum bond length. The interactions between non-bonded points is described by the Lennard-Jones potential (LJ)

$$U_{LJ} = \begin{cases} 4\varepsilon\left[\left(\frac{\sigma}{r_{ij}}\right)^{12} - \left(\frac{\sigma}{r_{ij}}\right)^{6}\right] - C, & r_{ij} \le r_c \\ 0, & r_{ij} > r_c \end{cases} \tag{S18}$$

where ε is the quenching depth, σ is the dameter of the monomers, r_{ij} is the distance between monomer i and j, r_c is the cutoff distance and $C = 4\varepsilon[(\sigma/r_c)^{12} - (\sigma/r_c)^6]$. To study the collapse dynamics, microgels are first simulated in good solvent conditions, where $r_c = 2^{1/6}\sigma$, untill reaching equilibrium and then the cutoff distance is set to $r_c = 2.5\sigma$ to model the poor solvent condition. The coupling between the MPC fluid and the monomers is achieved in the collision step *(49)*. Newton's equations of motion for the microgel are integrated by the velocity Verlet algorithm with a time step Δt, which is smaller than the collision-time step h.

The size of the microgel is characterized by its radius of gyration R_g, which is defined as

$$R_g^2(t) = \frac{1}{N}\sum_{i=1}^{N}\left(\boldsymbol{r}_i(t) - \boldsymbol{r}_{cm}(t)\right)^2 \tag{S19}$$

where $\boldsymbol{r}_{cm} = \frac{1}{N}\sum_{i=1}^{N}\boldsymbol{r}_i$ is the center of mass position of the microgel and N is the total number of monomers in the microgel.

section 2.3. Parameters

We consider microgels comprised of subchains with $N_m = 20$, 30, and 40 monomers and 729 crosslinks with 64 dangling ends at the outer surface of the microgel. By choosing l, k_BT, and m as units of length, energy, and mass, the unit of time is $\tau = (ml^2/k_BT)^{0.5}$. In addition, we set $\sigma = 0.8l$, $a = l$, $M = 10m$, $\bar{\alpha} = 130°$, the average number of fluid particles in a collision cell $\langle N_s \rangle$ to $\langle N_s \rangle = 10$, and $K = 10^3\ k_BT/l^2$. The collision time step is $h = 0.1\ \tau$, and we perform 20 MD simulation steps between collision steps.

section 2.4. Effect of the hydrodynamic interactions

The radius of gyration of the polymers during collapse follows the relation $\langle R_g^2(t) \rangle = \langle R_g^2(0) \rangle - At^{\alpha}$. In order to obtain the exponent α, $\langle R_g^2(0) \rangle - \langle R_g^2(t) \rangle$ is plotted versus time in fig. S18. The exponent α for each curve is obtained by fitting a power-law function in time regime $10 < t/\tau < 300$. The obtained exponent is $\alpha = 1.08 \pm 0.05$, i.e., the microgel size decreases approximately linearly with time.

section 2.5. Effect of cononsolvent transport

The cononsolvent transport into the microgel is described in a coarse-grained manner. Thereby, an imaginary sphere, centered at the center of mass of the microgel, is assumed around the microgel. Then, the radius of the sphere is reduced with a speed v_{cosol} and all the monomers outside the sphere are marked as attractive (poor solvent condition), while the monomers inside the sphere are treated as being in good solvent condition. The collapse speed of the microgels v_{mg} is determined by measuring the slope of the collapse curves (cf. fig. S19(a)) at the time, where the

radius of gyration radius meets the condition $R_g(t) = R_g(0)/2$. Figure S19(b) shows the normalized collapse speed of a microgel versus the normalized transport speed. It should be noted that v_0 is the intrinsic collapse speed of the microgel for infinitely fast transport of the cononsolvent into the microgel (i.e., the quenching depth, ε, instantaneously changes for all monomers); the transport depends on the network structure and the quenching depth. Figure S19 suggests that if the transport of cononsolvent particles is faster that the intrinsic collapse speed of the microgel (i.e., $v_{cosol}/v_0 > 1$), then the initial fast collapse regime becomes independent of the diffusion process. However, if the transport speed of cononsolvent particles is slower that the intrinsic collapse speed of the microgel (i.e. $v_{cosol}/v_0 < 1$), the microgel collapse speed is defined by the speed of the cononsolvent transport.

section 2.6. Evolution of structure during microgel collapse for low quenching depth

The radial distribution function of monomers during the microgel collapse for a low quenching depth is shown in fig. S20(a). The figure, together with snapshots in fig. S20(b), show the structural evolution from early stages of the collapse, where small clusters are formed near the crosslinking sites ($t = 100\tau$), to the formation of a hollow core-shell-like structure (i.e., $t = 400\tau$), which, at the end, turns into a collapsed globule ($t = 2000\tau$). To characterize the core-shell-like structure, we determine the total radius $R_T(t)$ of the microgel and the thickness of its core $W_{core}(t)$, where these quantities are defined as the maximum and minimum radius at which the radial density distribution assumes half of the value of its maximum. The thickness of the shell is correspondingly defined as $W_{shell}(t) = R_T(t) - W_{core}(t)$. The thickness analysis (inset) shows that the structural changes in the core region of the microgel continue while the overall size of the particle does not show a considerable change anymore ($t/\tau > 1250$).

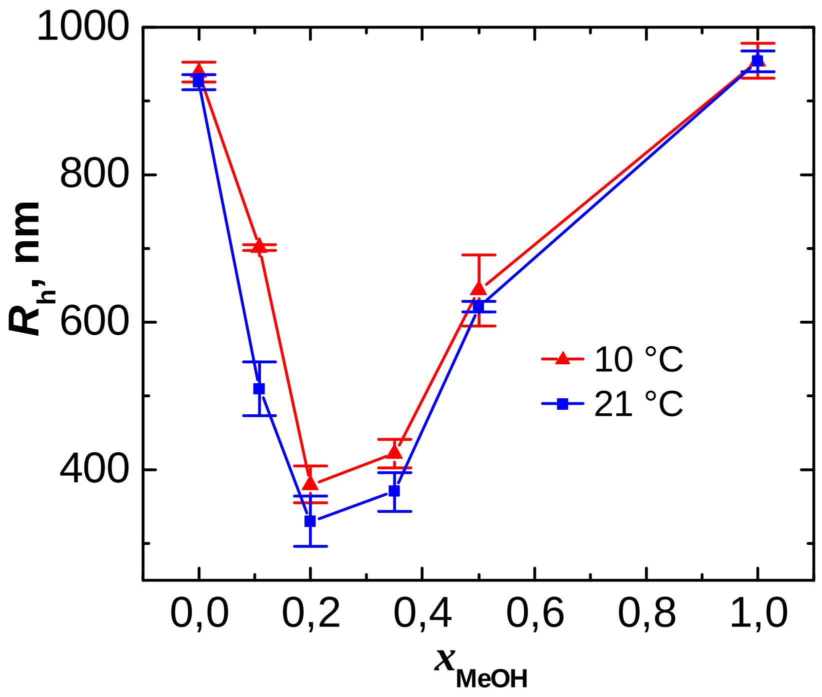

fig. S1. Hydrodynamic radius as a function of the methanol mole fraction at 10° and 21°C.

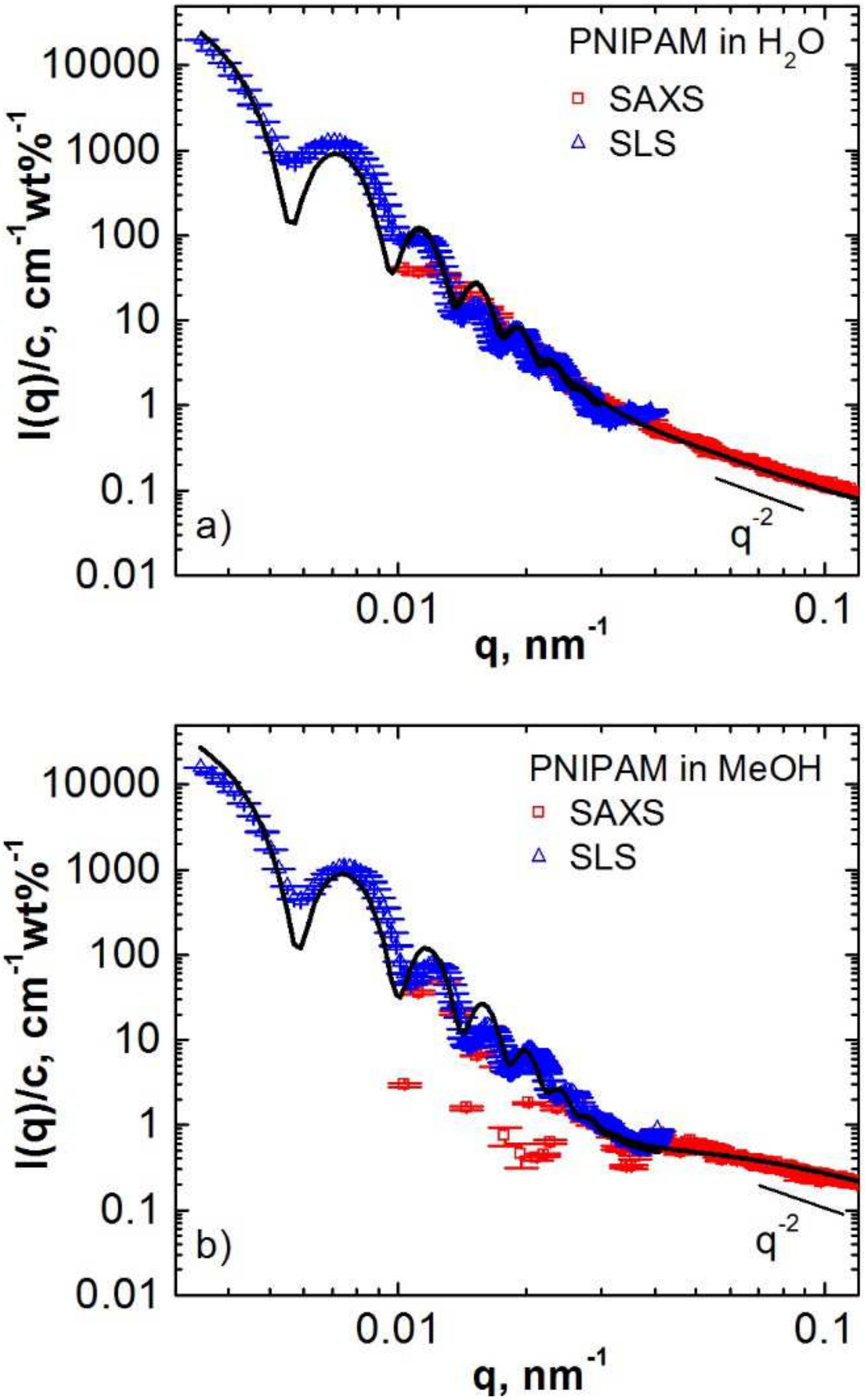

fig. S2. Normalized scattering curves for PNIPAM microgel at 10°C measured by SLS in the small *q* range and SAXS in the high q range. (a) pure H_2O and (b) pure MeOH. Solid lines represent fits according to the model described by Stieger et. al. *(37).*

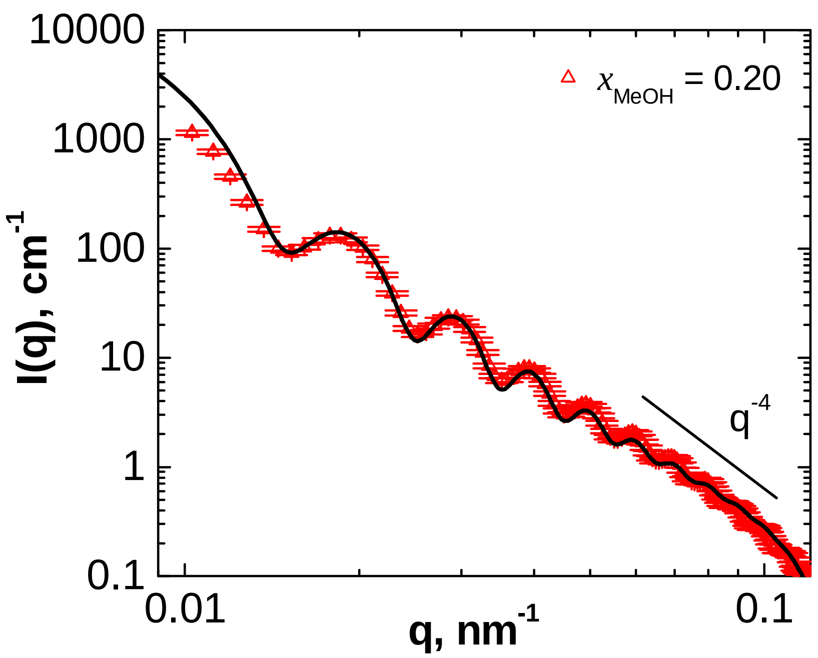

fig. S3. Static SAXS curves for PNIPAM microgel in x_{MeOH} = 0.20 at 10°C. Concentration of samples is 0.40 wt%. The solid line is the fitting curve obtained with the model described by Stieger et. al. *(37).*

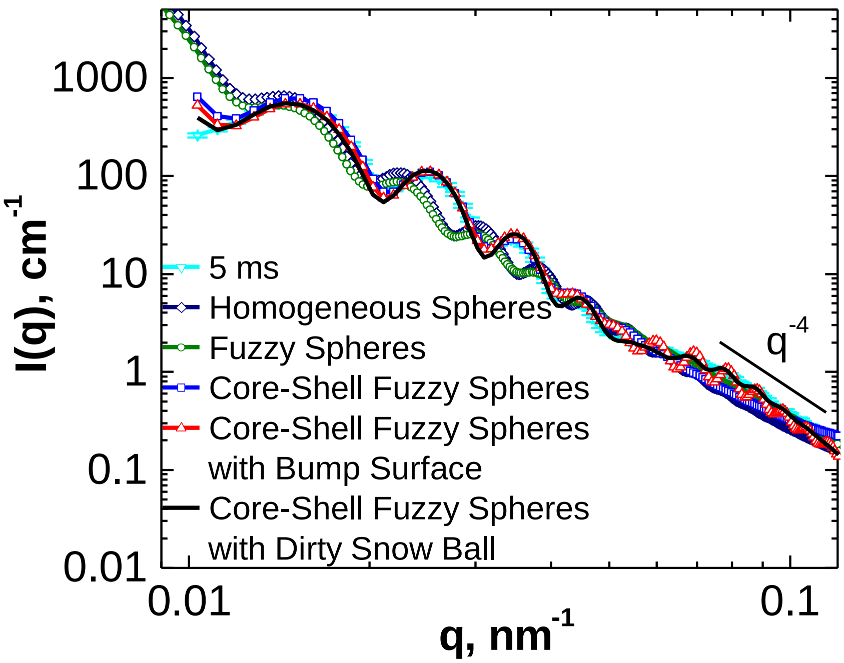

fig. S4. Radially averaged SAXS pattern for PNIPAM in the solvent composition jump from pure MeOH to x_{MeOH} = 0.20 at 5 ms after the mixture. Solid lines represent the fits obtained with the following models: Homogeneous sphere (blue line), fuzzy sphere l (green line), simple core-shell fuzzy sphere (gray line), core-shell fuzzy sphere with small collapsed regions located at the surface (red line) and core-shell fuzzy spheres with distributed collapsed regions/clusters (black line).

Additional information about the jump from MeOH

Fitted TR-SAXS scattering curves at different times

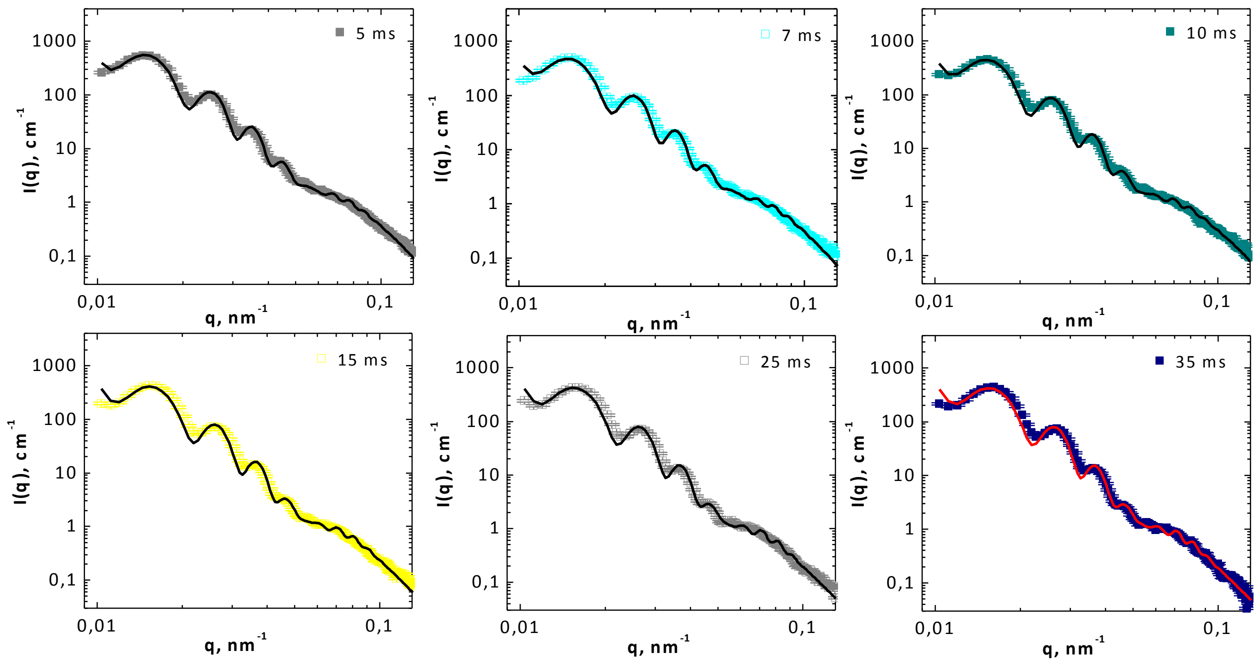

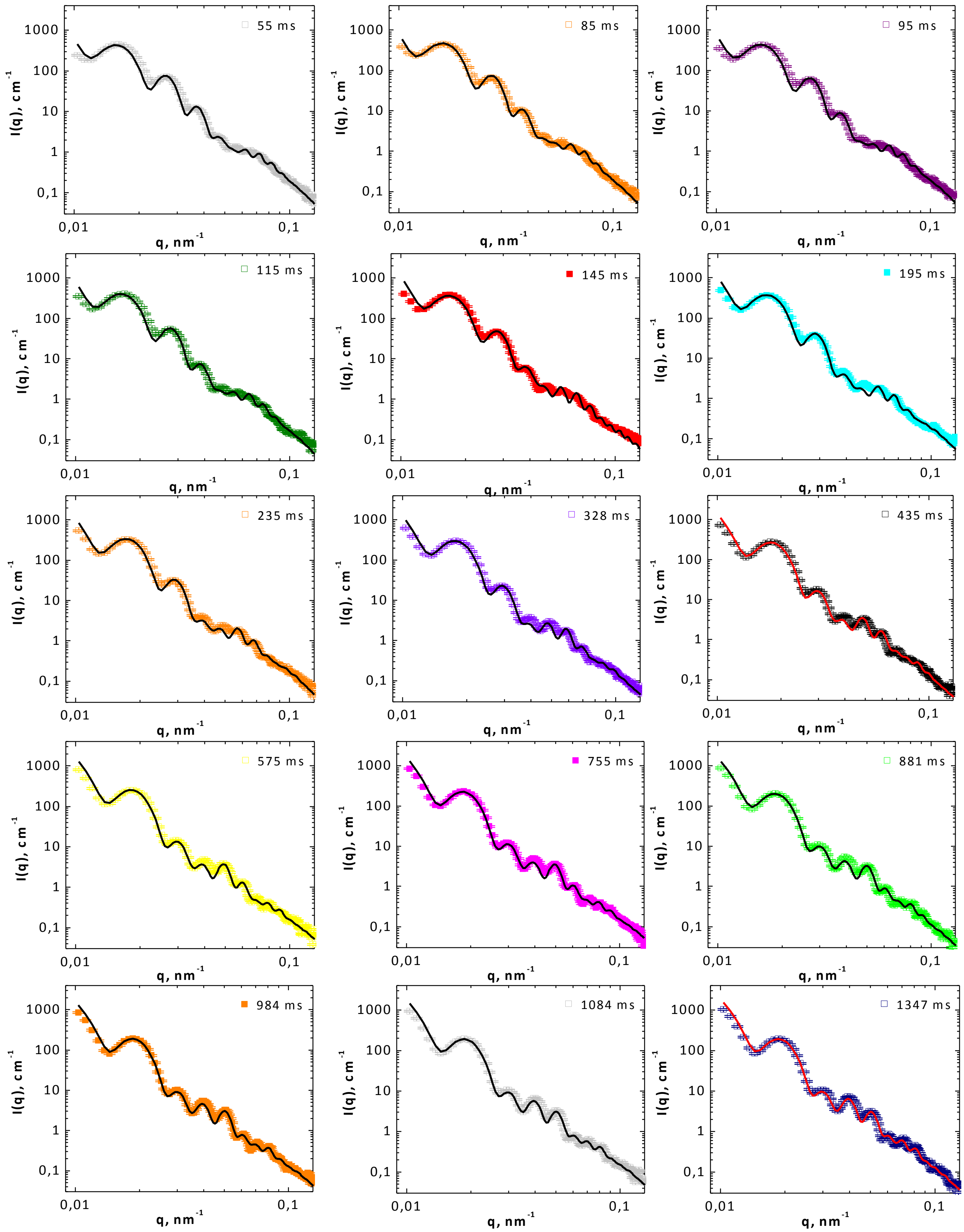
55 ms
85 ms
95 ms
115 ms
145 ms
195 ms
235 ms
328 ms
435 ms
575 ms
755 ms
881 ms
984 ms
1084 ms
1347 ms
I(q), cm-1
q, nm-1
1000
100
10
1
0,1
0,01
0,1

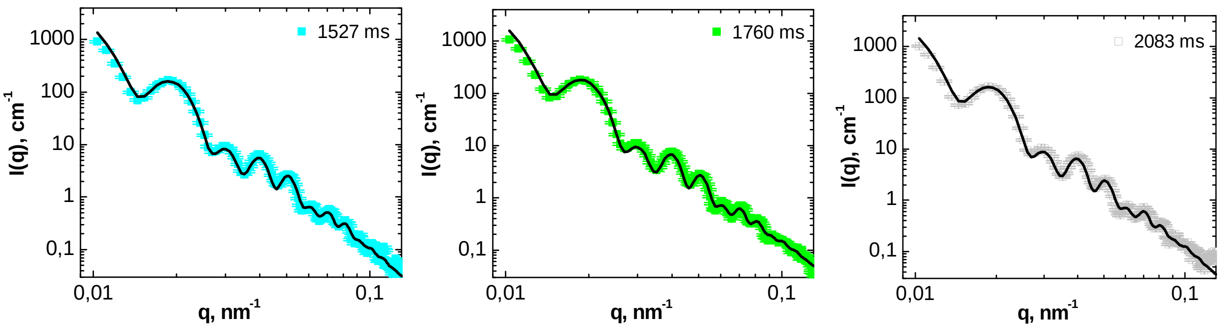

fig. S5. Radially averaged SAXS and fit curves for PNIPAM microgel in the solvent composition jump from pure MeOH to x_{MeOH} = 0.20. Solid lines represent the fits obtained with the model of core-shell fuzzy spheres including distributed collapsed regions/clusters.

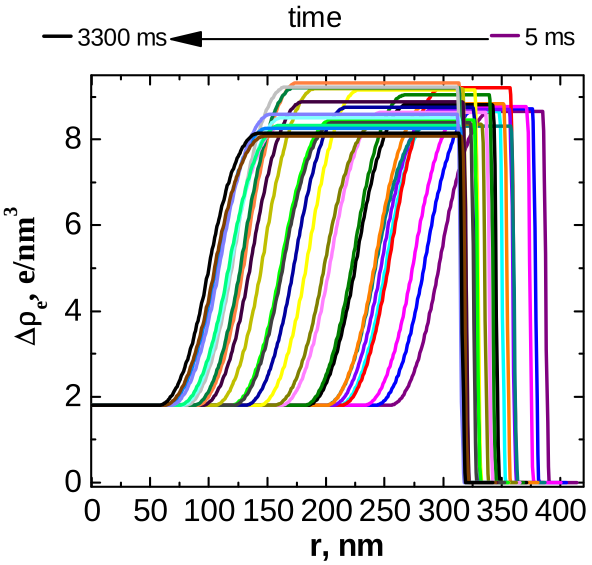

fig. S6. Radial excess electron density profiles calculated from the modeling procedure for PNIPAM microgels in the solvent composition jump from MeOH to x_{MeOH} = 0.20 at 10°C.

Information about jump from water

Scattering curves

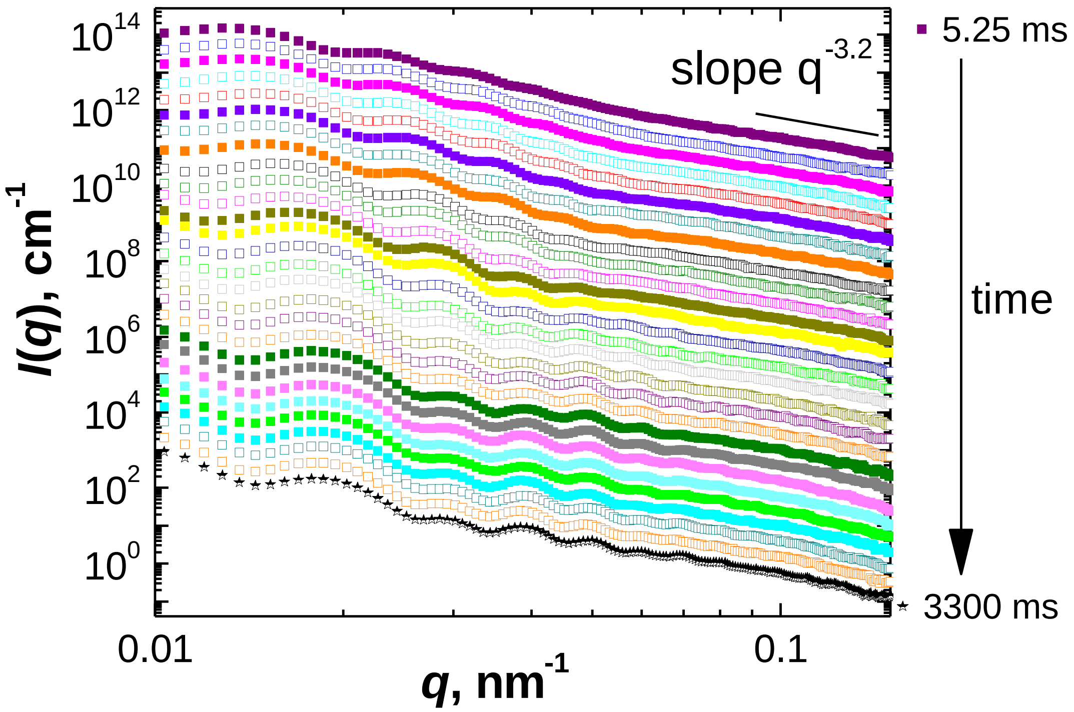

fig. S7. Radially averaged SAXS patterns of PNIPAM microgels for the solvent composition change from pure H2O to x_{MeOH} = 0.20.

Fitted TR-SAXS scattering curves for all points in time

TR-SAXS: Solvent composition jump from pure H_2O to x_{MeOH} = 0.20

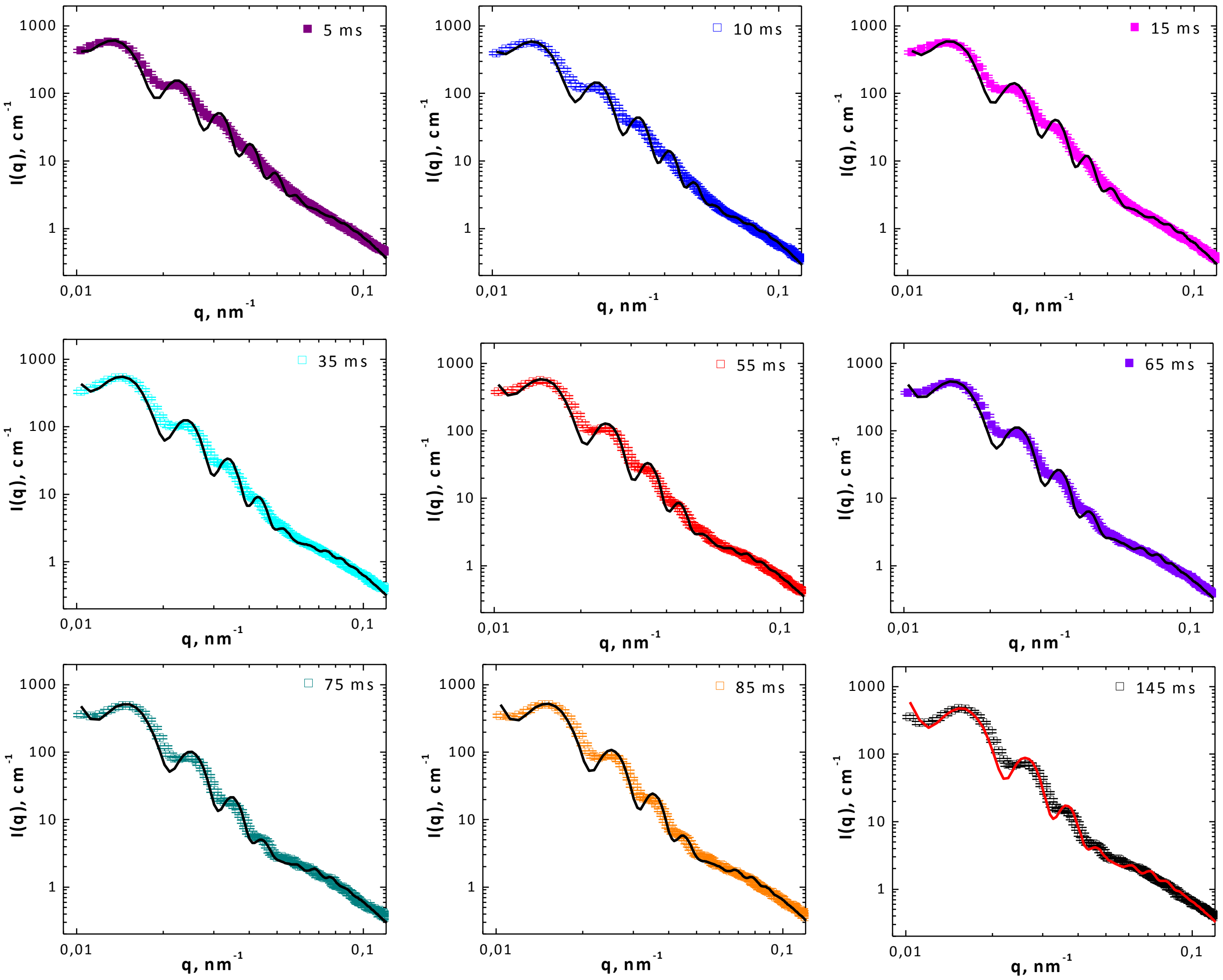

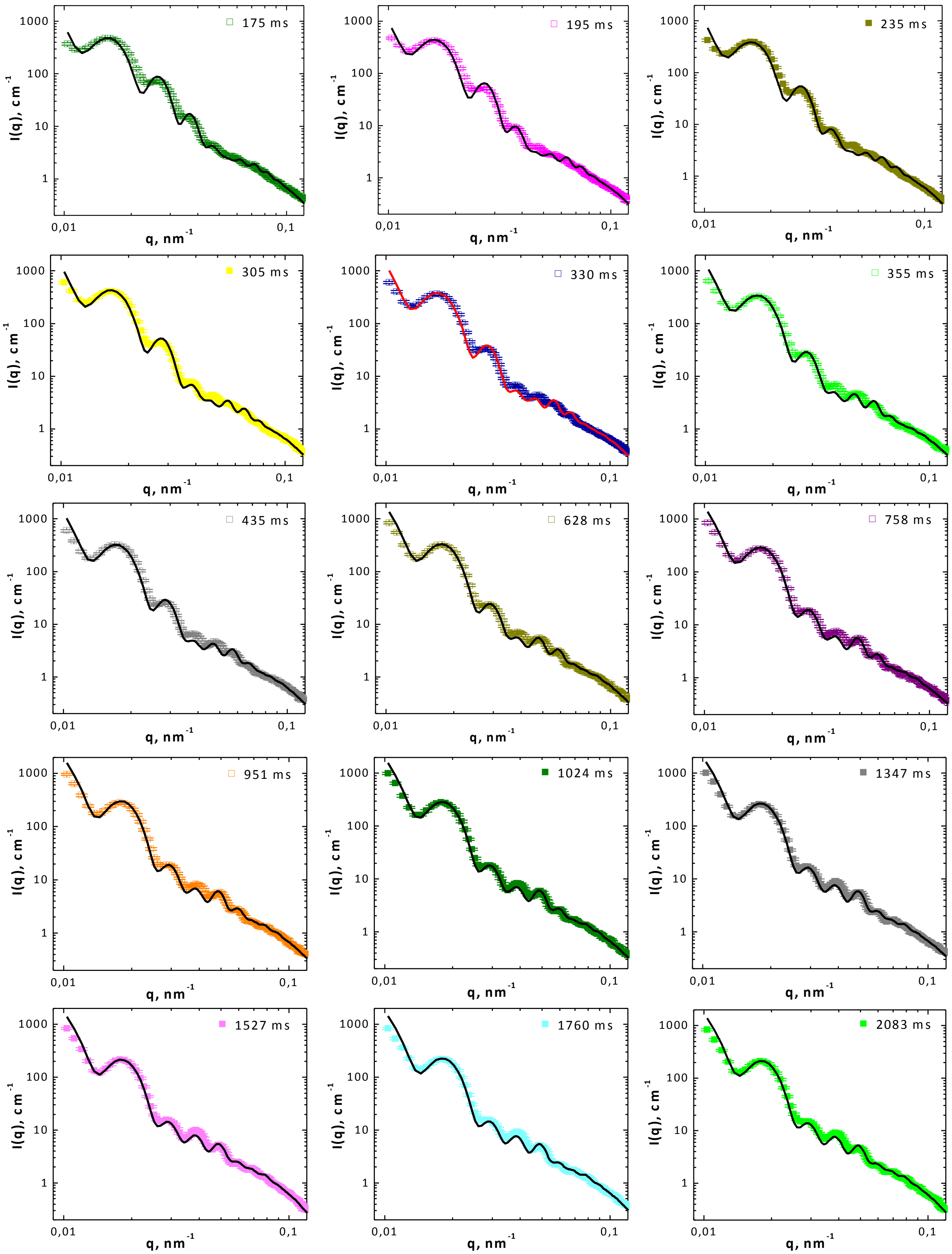

fig. S8. Radially averaged SAXS patterns and fit curves for PNIPAM in the solvent composition jump from pure H_2O to x_{MeOH} = 0.20. Solid lines represent the fits obtained with the model of core-shell fuzzy spheres. The overall behavior is also excellently reproduced by the model except for the depth of the minima at very short times.

This could be due to problems in estimating the width of the resolution function, polydispersity, and also due to possible minor deviations from spherical symmetry, which are not included in the model.

Radial excess electron densities

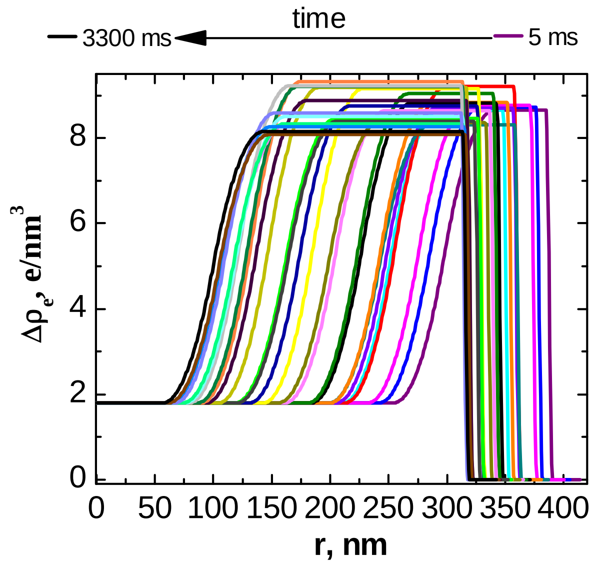

fig. S9. Radial excess electron density profiles calculated from the modeling procedure for PNIPAM microgels in the solvent composition jump from H2O to x_{MeOH} = 0.20 at 10°C.

Size evolution

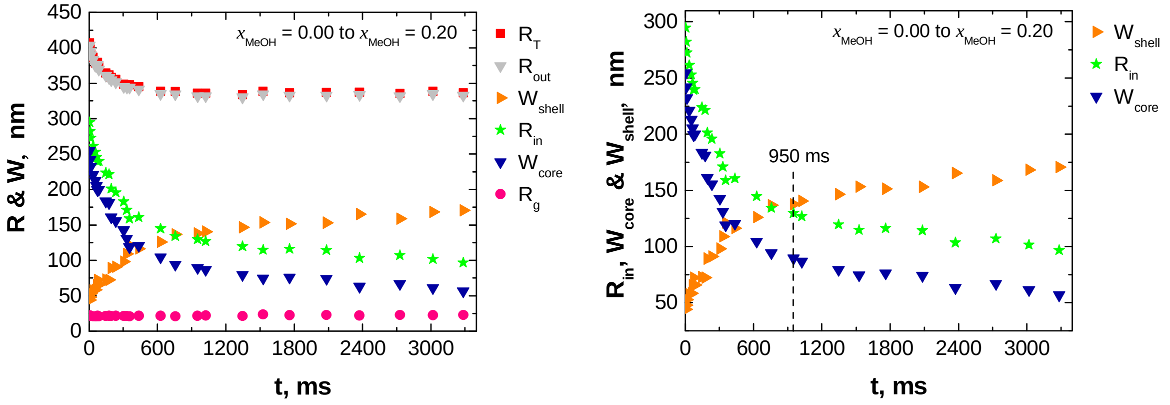

fig. S10. Fit results for the collapse transition of PNIPAM induced by the solvent composition jump from pure H_2O to x_{MeOH} = 0.20 at 10°C.

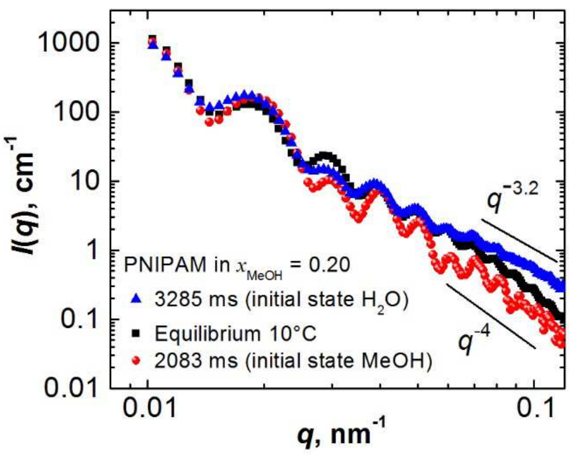

fig. S11. SAXS curves of PNIPAM in x_{MeOH} = 0.20 obtained by the static equilibrium measurements (squares), by the solvent composition change from MeOH (circles), and by the solvent composition change from H_2O (triangles). A slope of -4 is observed for collapsed particles with sharp surface. The data obtained for the jump from pure water after 3285 ms reveal deviations, indicating that final state has not yet been reached after this time.

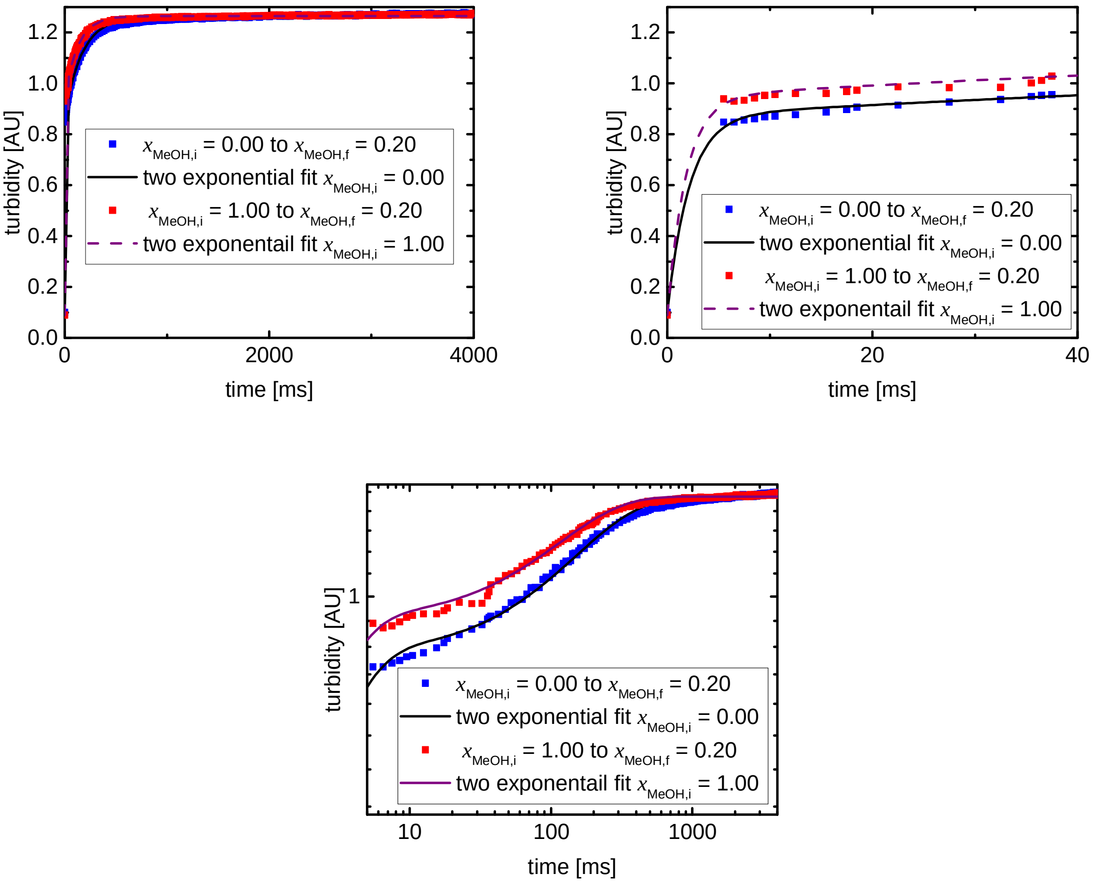

fig. S12. Turbidity as a function of time for the collapse transition of PNIPAM microgel induced by changing the solvent composition from pure solvent (either H_2O or MeOH) to x_{MeOH} = 0.20 at 10°C. (top left) turbidity over time; (top right) Zoom to short times and fit with exponential functions and (bottom) corresponding log-log plot.

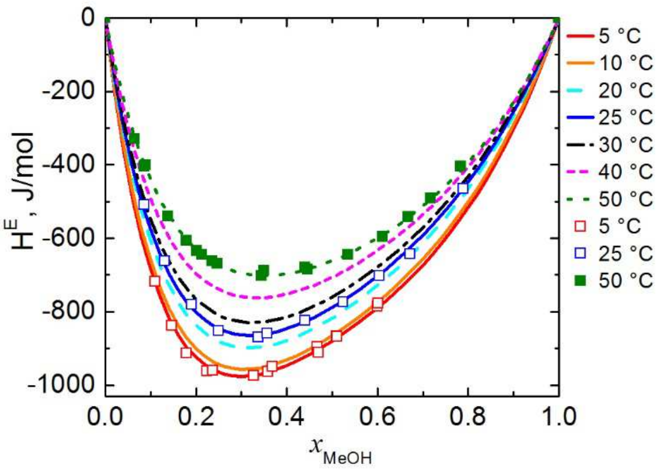

fig. S13. Effect of the temperature on the excess enthalpy H^E of mixing H_2O and MeOH. Solid lines represent the theoretical estimations, while the dots correspond to the experimental data from reference *(71)*.

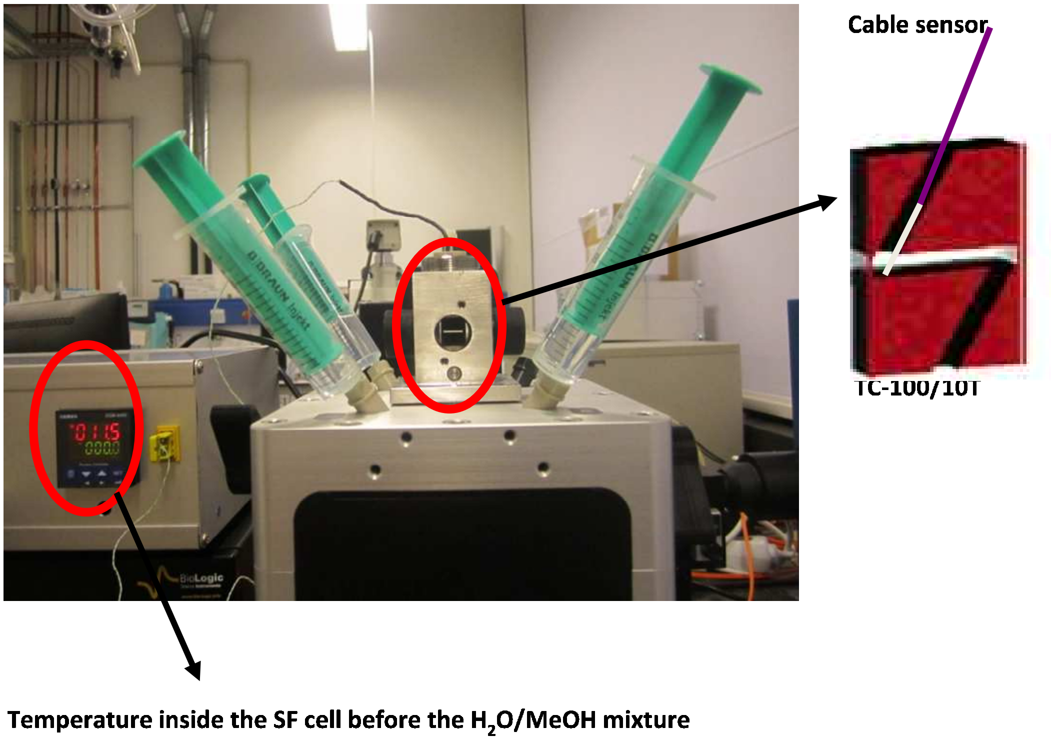

fig. S14. Schematic representation of the stopped-flow setup for the estimation of the increase of the temperature inside the TC-100/10T cuvette upon H_2O/MeOH mixing.

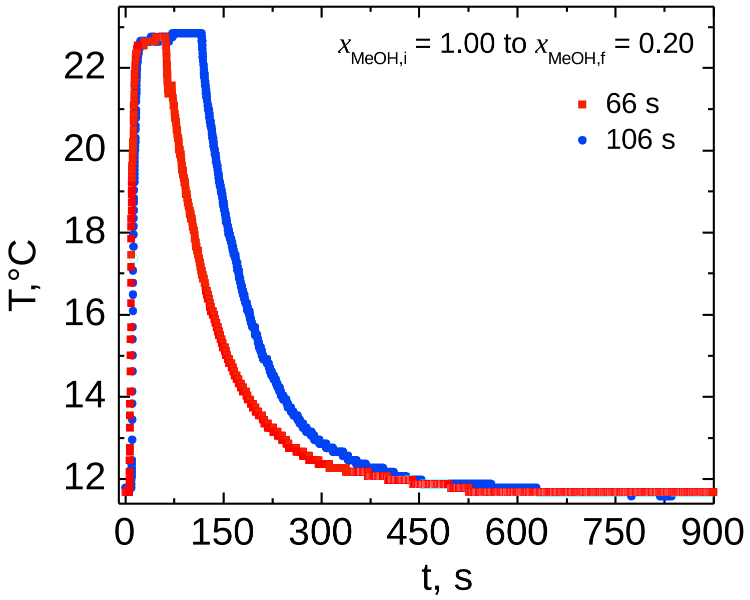

fig. S15. Increase of the temperature inside the TC-100/10T cuvette with time by mixing H_2O and MeOH at 10°C to reach a final solvent composition of x_{MeOH} = 0.20. The mixture flows through the cuvette for 66 s (red) and for 106 s (blue).

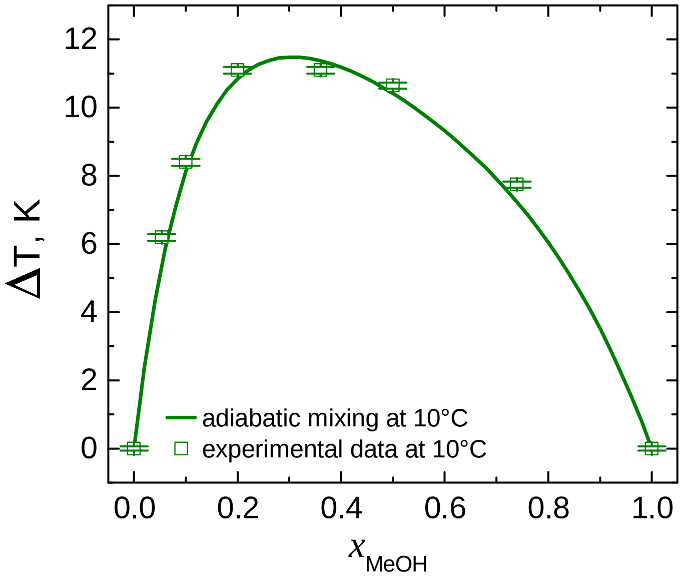

fig. S16. Increase of the temperature inside the TC-100/10T cuvette during the H_2O/MeOH mixing.

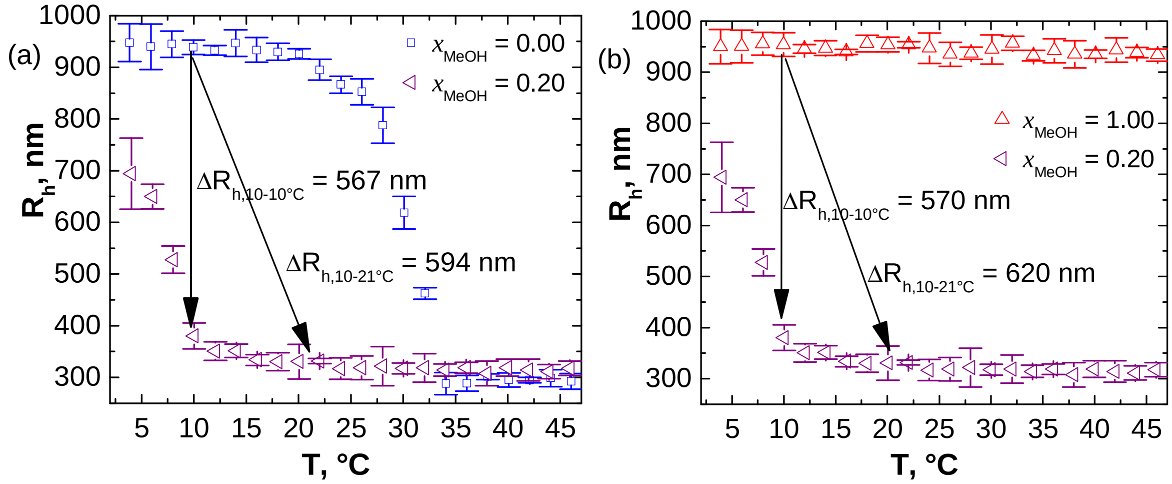

fig. S17. Comparison of the temperature-dependent size of PNIPAM microgel. (a) in pure H_2O and $x_{MeOH} = 0.20$; and (b) in pure MeOH and $x_{MeOH} = 0.20$. The arrows indicate the solvent composition jump from pure MeOH to $x_{MeOH} = 0.20$ assuming either no increase or an increase in temperature due to the mixing enthalpy H^E of H_2O/MeOH.

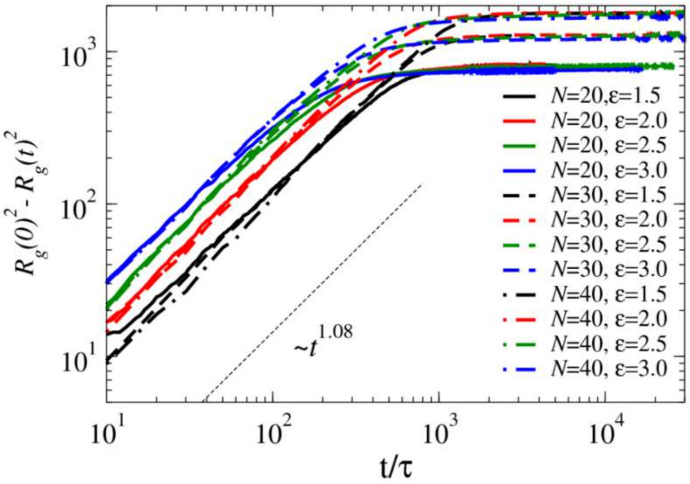

fig. S18. Simulation results of the time evolution of $\langle \mathbf{R_g^2(0)} \rangle - \langle \mathbf{R_g^2(t)} \rangle$ for microgels with different quenching depths $\boldsymbol{\varepsilon}$ and different polymer lengths $\boldsymbol{N_\mathrm{m}}$.

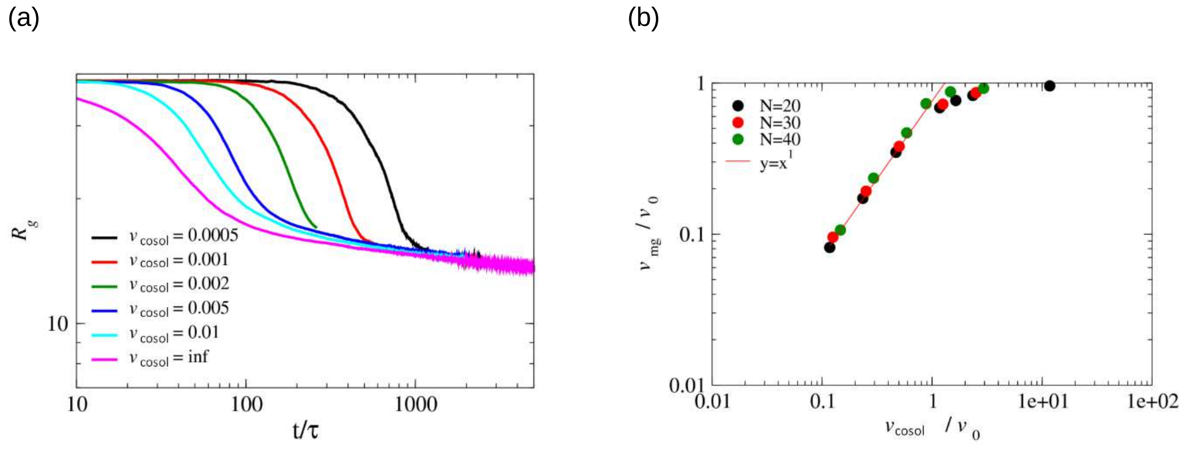

fig. S19. Results from simulations for evolution of the microgel size and collapse velocity. Simulation results of (**a**) the time evolution of the microgel radius of gyration R_g for various cononsolvent-transport velocities v_{cosol}, and (**b**) the normalized collapse velocity of microgels as function of the normalized transport velocity of the cononsolvent for various polymer lengths N_m. The quenching depths is $\varepsilon = 2.5$.

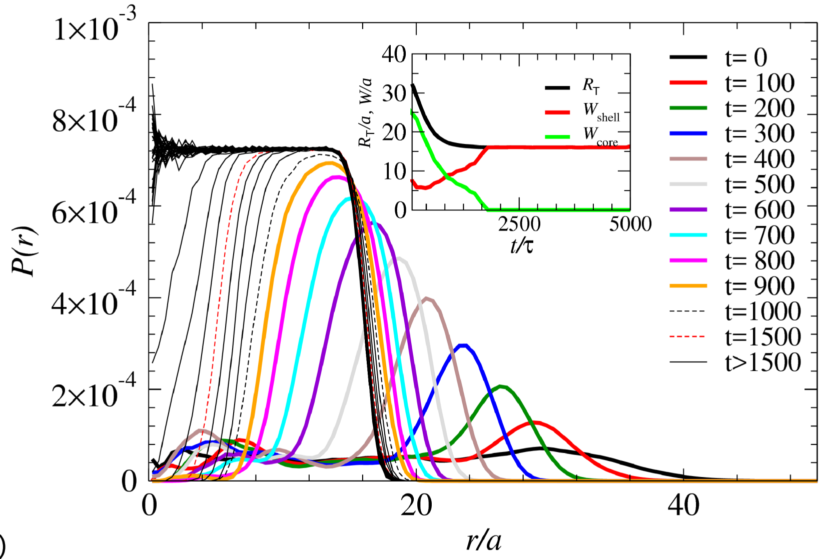

(a)

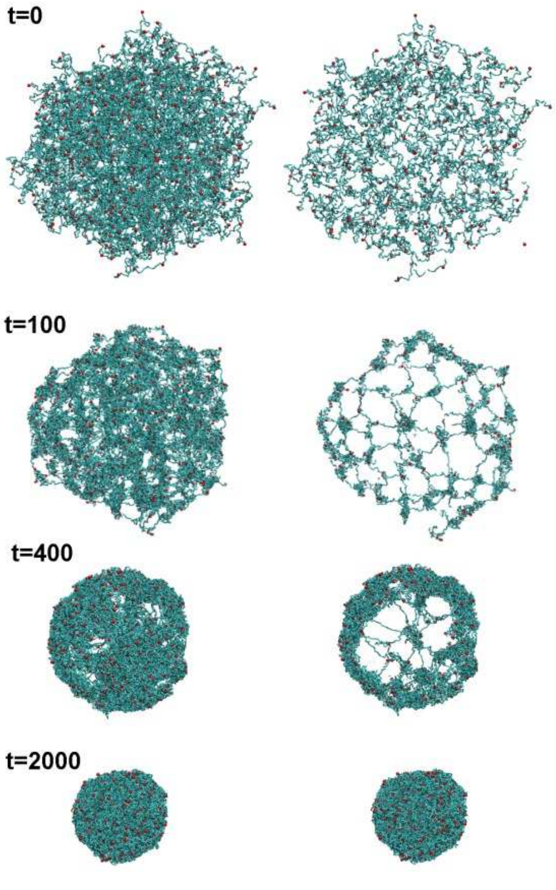

(b)

fig. S20. Results from simulations for monomer distribution and microgel conformations. (**a**) Simulation results for the radial monomer density distribution for a microgel with $N_m = 20$ and ε = 1.5. The inset shows the time

evolution of the outer radius $\boldsymbol{R_o}$, shell thickness $\boldsymbol{W_{shell}}$, and core thickness $\boldsymbol{W_{core}}$, respectively. (**b**) Snapshots of microgel conformations (thin slice through the center is shown on the right) at various times during the simulation.

table S1. Fit results for PNIPAM microgel in pure H_2O, pure MeOH, and x_{MeOH} = 0.20 at 10°C.

	***T*, °C**	***R*, nm**	σ_{surf}, nm	**R_T, nm**
**H_2O (SLS & SAXS)**	10	799	46	891
**MeOH (SLS & SAXS)**	10	770	45	860
**x_{MeOH} = 0.20 (SAXS)**	10	312	1.6	315
